# Evaluating the impact of age on immune checkpoint therapy biomarkers

**DOI:** 10.1016/j.celrep.2021.109599

**Published:** 2021-08-24

**Authors:** Rossin Erbe, Zheyu Wang, Sharon Wu, Joanne Xiu, Neeha Zaidi, Jennifer La, David Tuck, Nathanael Fillmore, Nicolas A. Giraldo, Michael Topper, Stephen Baylin, Marc Lippman, Claudine Isaacs, Reva Basho, Ilya Serebriiskii, Heinz-Josef Lenz, Igor Astsaturov, John Marshall, Josephine Taverna, Jerry Lee, Elizabeth M. Jaffee, Evanthia T. Roussos Torres, Ashani Weeraratna, Hariharan Easwaran, Elana J. Fertig

**Affiliations:** 1 McKusick-Nathans Institute of the Department of Genetic Medicine, Johns Hopkins School of Medicine, Baltimore, MD, USA; 2 Department of Oncology, Sidney Kimmel Comprehensive Cancer Center, Johns Hopkins School of Medicine, Baltimore, MD, USA; 3 Department of Biostatistics, Johns Hopkins Bloomberg School of Public Health, Baltimore, MD, USA; 4 Caris Life Sciences, Irving, TX, USA; 5 VA Boston Healthcare System, Boston, MA, USA; 6 Department of Dermatology, Johns Hopkins University School of Medicine, Baltimore, MD 21287, USA; 7 Lombardi Comprehensive Cancer Center, Georgetown University Medical Center, Washington, DC, USA; 8 Cedars-Sinai Medical Center, Samuel Oschin Comprehensive Cancer Institute, 8700 Beverly Boulevard, #AC-1046A, Los Angeles, CA 90048, USA; 9 Fox Chase Cancer Center, Philadelphia, PA, USA; 10 Keck School of Medicine, University of Southern California, Los Angeles, CA, USA; 11 Division of Hematology and Oncology, Department of Medicine, University of Texas Health Science Center, San Antonio, TX, USA; 12 Department of Biochemistry and Molecular Biology, Johns Hopkins Bloomberg School of Public Health, Baltimore, MD, USA; 13 Department of Applied Mathematics and Statistics, Johns Hopkins University Whiting School of Engineering, Baltimore, MD, USA; 14 Department of Biomedical Engineering, Johns Hopkins Bloomberg School of Medicine, Baltimore, MD, USA; 15 Lead contact

## Abstract

Both tumors and aging alter the immune landscape of tissues. These interactions may play an important role in tumor progression among elderly patients and may suggest considerations for patient care. We leverage large-scale genomic and clinical databases to perform comprehensive comparative analysis of molecular and cellular markers of immune checkpoint blockade (ICB) response with patient age. These analyses demonstrate that aging is associated with increased tumor mutational burden, increased expression and decreased promoter methylation of immune checkpoint genes, and increased interferon gamma signaling in older patients in many cancer types studied, all of which are expected to promote ICB efficacy. Concurrently, we observe age-related alterations that might be expected to reduce ICB efficacy, such as decreases in T cell receptor diversity. Altogether, these changes suggest the capacity for robust ICB response in many older patients, which may warrant large-scale prospective study on ICB therapies among patients of advanced age.

## In brief

Erbe et al. use multi-omics databases to evaluate biomarkers that are used to predict the response of patients with cancer to ICB in the context of aging. Erbe et al. find that biomarkers associated with ICB response are enriched in tumors from older patients relative to their younger counterparts.

## Graphical Abstract

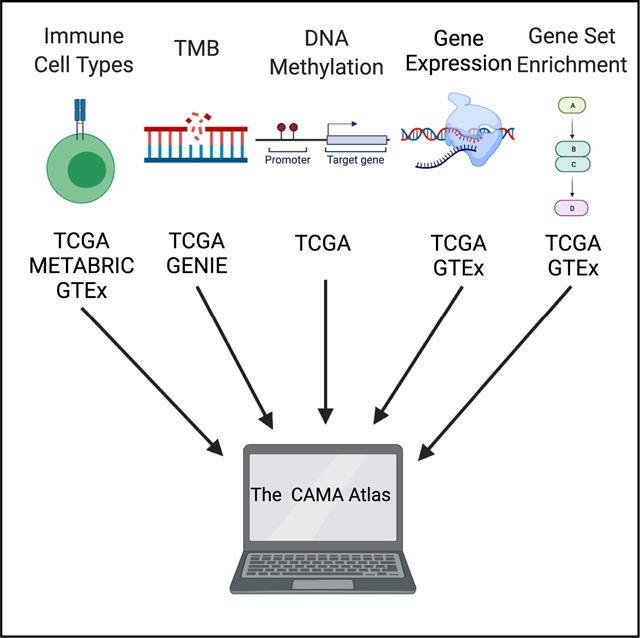

## INTRODUCTION

The association of cancer incidence with age is well established, and the phenomenon of age-related immune decline has been recognized for even longer (Gardner, 1980). Mutations and DNA methylation have been shown to accumulate with age and drive carcinogenesis (Tomasetti et al., 2017; Horvath, 2013; Klutstein et al., 2017; Xie et al., 2018). Recent research has highlighted the specific changes that contribute to the general decline of the immune system that occurs as individuals age (Aw et al., 2007). Understanding the effect such alterations have on the anti-tumor immune response is critical for the informed development and application of immunotherapies to elderly patients.

Outside the context of cancer, older individuals are generally observed to have less effective immune responses to disease (Gardner, 1980). This observation is commonly associated with systemic immune aging. In particular, loss of T cell receptor (TCR) diversity (Britanova et al., 2014), decreased capacity of cytotoxic cells (Solana and Mariani, 2000), and increased inflammatory signaling (Franceschi et al., 2000) have been identified as age-related immune changes. These studies note the potential significance of these forms of immune aging on cancer, and indeed systemic immune aging has received considerable attention in the context of its effect on cancer development and progression (Fulop et al., 2018). Still, the potential translation of these findings to cancer therapeutics and patient care requires further comprehensive evaluation of the interplay between systemic immunity and the tumor immune microenvironment resulting from aging, particularly in the context of immunotherapy. In spite of the general immune decline associated with aging, the majority of clinical trial analyses suggest that elderly patients experience no reduced benefit or even increased benefit as compared with younger patients on immune checkpoint blockade (ICB) therapies (Kugel et al., 2018; Elias et al., 2018; Jain et al., 2020). However, there is still some contention on this point (Daste et al., 2017), and elderly patients are less likely to be treated with ICB therapies than their younger counterparts (Hurez et al., 2018; Jain et al., 2020).

High-throughput molecular data from atlas studies provide new opportunities to comprehensively characterize the immune landscape of tumors (Thorsson et al., 2018) and are now sufficiently powered to evaluate aging-related changes (Wu et al., 2019; Shah et al., 2020; Chatsirisupachai et al., 2021). This study leverages genomics and clinical data from 9,523 patients across 31 cancer types from The Cancer Genome Atlas (TCGA); 37,961 patients across 8 cancer types from the Genomics Evidence Neoplasia Information Exchange (GENIE); 15,557 patients with breast, colon, or head and neck cancers from Caris Life Sciences (CLS); 1,818 patients with breast cancer from Molecular Taxonomy of Breast Cancer International Consortium (METABRIC); and genomics data from a pan-tissue reference of 948 non-cancer individuals from the Genotype-Tissue Expression (GTEx) Project (see Supplemental Data S1 for a summary of patient characteristics in each cohort) to evaluate such age-related changes in the tumor immune landscape. Because the immune microenvironment mediates ICB response, we focus our analysis of these large-scale data to evaluate the impact of aging on the molecular and cellular biomarkers of ICB response, such as *PDL1* expression (Patel and Kurzrock, 2015), tumor mutational burden (TMB) (Yarchoan et al., 2017), (Goodman et al., 2017), cell-type composition of the immune tumor microenvironment (ITME) (Frankel et al., 2017), TCR diversity (Han et al., 2020), expression of other immune checkpoint genes (Taube, 2014), and expression of inflammation-related pathways, such as interferon gamma (Cristescu et al., 2018; Higgs et al., 2018) and transforming growth factor β (TGF-β) signaling (Tauriello et al., 2018). We further compile these analyses into a web application, Cancer Associations with Molecular Aging (CAMA), to allow for further customized analyses of the cellular and molecular pathways altered with age pan-cancer. Our analyses from CAMA in the context of ICB biomarkers suggest that the aged ITME upregulates major pathways associated with immune response, although additional indicators of immune decline warrant future prospective clinical studies to provide databases of combined genomics and clinical data in order to directly evaluate the impact of age on the ITME in the context of ICB response.

## RESULTS

### TMB increases with age in most cancers, while TCR diversity decreases

**Figure 1.**
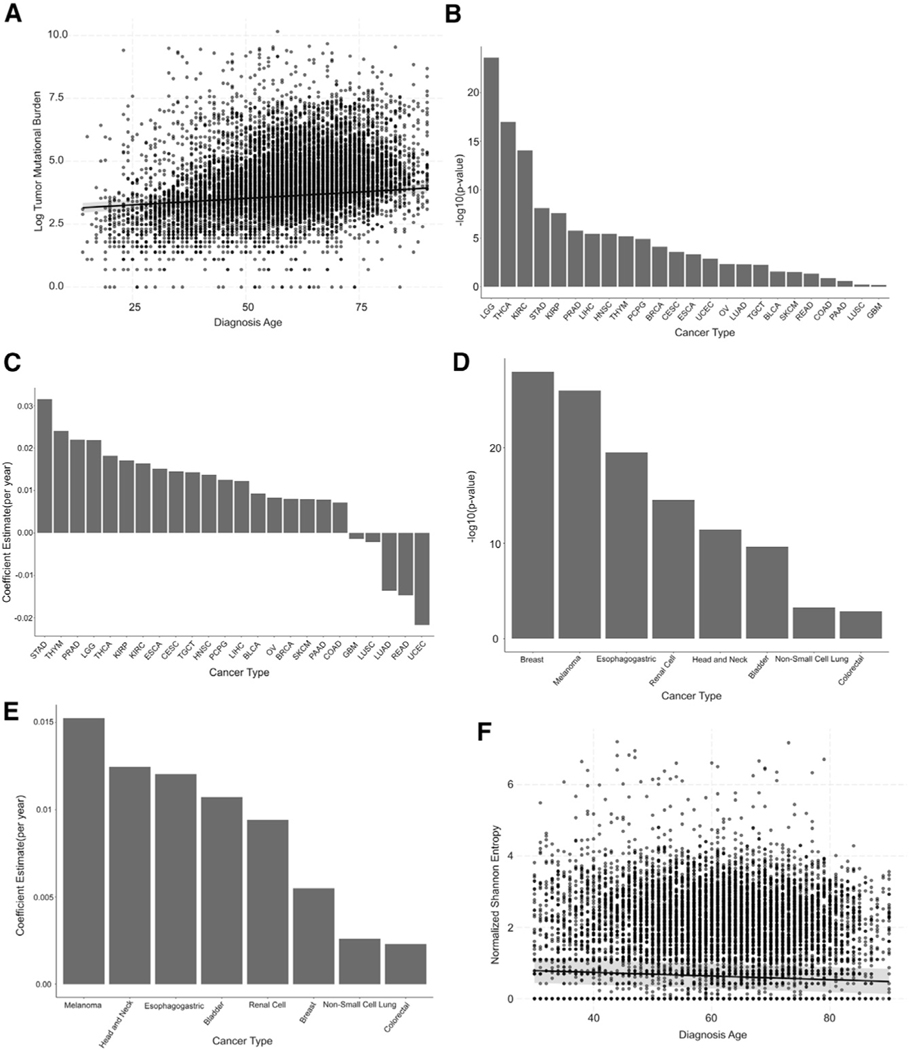
TMB generally increases and TCR diversity decreases with patient age at diagnosis (A) Scatterplot of log2 tumor mutational burden by patient diagnosis age pan-cancer in TCGA data. The linear trend predicted by a multivariate linear model that includes cancer type as a covariate is shown. (B) Bar plot of the negative log10 p values for the age term of linear models fit for TMB in each TCGA cancer type study. (C) Bar plot of the coefficient estimates per year for the age term of linear models fit for each TCGA cancer type study. Positive coefficients indicate increased mutational burden with increasing age. (D) Bar plot of the negative log10 p values for the age term of linear models fit for TMB within eight cancer types commonly treated with ICB therapies in data from GENIE. (E) Bar plot of the coefficient estimates per year for the age term of linear models fit for TMB within eight cancer types commonly treated with ICB therapies in data from GENIE. (F) Scatterplot of normalized Shannon entropy of TCR sequences by patient diagnosis age pan-cancer in TCGA data. The linear trend predicted by a multivariate linear model that includes cancer type as a covariate is shown.

The large number of public domain genomics datasets from primary tumors and normal tissue in the literature provides the opportunity to characterize the impact of age on the ITME and ICB biomarkers. Due to the widespread use of TMB as a primary clinical biomarker of ICB therapy (Yarchoan et al., 2017; Goodman et al., 2017), we first examine the relationship of TMB with patient age. As has been previously reported among TCGA samples (Chalmers et al., 2017; Qing et al., 2020), we find TMB significantly increases with patient age at diagnosis (1.02% increase per year of age, p < 1 × 10^−16^) (Figure 1A) pan-cancer in TCGA when modeling cancer type as a covariate. This association is further observed within most cancer types (Figures 1B and 1C), although both lung adenocarcinomas (−1.31% per year, q = 0.0072) and uterine carcinomas (−2.02% per year, q = 0.0022) demonstrate decreased TMB with age. To validate these findings, we also investigate the relationship of age and TMB within the larger mutational dataset provided by GENIE for eight ICB-approved cancer types. This analysis identifies a significant increase in TMB with age in all eight cohorts (Figures 1D and 1E). In contrast with TCGA, non-small cell lung cancer samples in the GENIE cohort show a small increase in TMB with age (0.2% increase per year, q = 6.13 × 10^−4^). We further identify significant increases in TMB among elderly CLS patients with colorectal and breast cancer (q = 4.92 × 10^−15^ and q = 6.39 × 10^−11^, respectively) (Figure S1). In contrast with TCGA and GENIE, the null hypothesis is not rejected in human papillomavirus (HPV)-negative head and neck cancers (q = 0.244) (Figure S1). These results provide a robust indication of TMB increases with age across most, if not all, ICB-approved cancer types.

The canonical interpretation of TMB as a biomarker for ICB therapy is that more mutations generally implies more immunogenic mutations, which in turn makes it more likely for an antigen to be displayed via major histocompatibility complex (MHC) class I that T cells are able to recognize, allowing them to target the corresponding tumor cells. Another factor in the likelihood of this recognition event is the number of antigens T cells infiltrating the tumor are able to recognize, defined by the TCR sequence carried by each T cell. The overall decline in the total number of unique TCR clones as part of the normal aging process (Yager et al., 2008; Britanova et al., 2014; Egorov et al., 2018) is well established in the literature. The process of thymic involution (the loss of thymus tissue with age) eventually ends the production of naive T cells and is the major driver of normal age-related decreases in T cell clonality (Aspinall and Andrew, 2000). However, the impact of carcinogenesis on age-related T cell clonality has not been fully characterized. To quantify aging-related changes in TCR clonality specific to the ITME, we leveraged estimates of TCR sequences previously generated with the miTCR algorithm (Bolotin et al., 2013) by Thorsson et al. (2018) from RNA sequencing (RNA-seq) data in TCGA to determine the association between TCR clonality and age. We define our metric of clonal diversity as the Shannon entropy multiplied by the number of unique clones divided by the total number of TCR sequencing reads to correct for variation in total number of T cells in each tumor sample. We determine that this TCR clonality measure significantly decreases with age for pan-cancer TCGA samples, including cancer type as a covariate (−0.0051 normalized Shannon entropy per year; p = 1.48 × 10^−8^) (Figure 1F), corresponding to a 0.26% predicted decrease per year in tumor TCR clonality relative to the mean normalized Shannon entropy of 1.95 observed with pan-cancer. Among individual cancer types, we observe a significant increase in TCR clonality with age in patients with lung adenocarcinoma (0.013 per year; q = 3.99 × 10^−3^) and significant decreases in patients with breast cancer (−0.01 per year; q = 2.63 × 10^−3^), uterine cancer (−0.012 per year; q = 0.011), melanoma (−0.015 per year; q = 3.99 × 10^−3^), and gastric cancer (−0.01 per year; q = 0.040) (Figure S2). These results indicate a general decrease in TCR clonality with age, although not necessarily a uniform one across cancer types.

### Age correlates with ICB-related gene expression among both patients with cancer and normal individuals

In addition to TMB and TCR clonality as biomarkers of ICB therapies, immune checkpoint gene expression can also be used as a biomarker for specific inhibitors. PDL1 expression is an established clinical biomarker to predict patient response to anti-PD1/PDL1 treatment (Patel and Kurzrock, 2015). More broadly, the efficacy of ICB immunotherapy is linked to the expression of target genes and their complementary receptors, such as *PD1, PDL1, CTLA4, CD80*, and *CD86* (Taube, 2014), as well as to associated genes, such as *PDL2, JAK2, LAG3, HAVCR2, TGFB1*, and *CXCL9* (Conway et al., 2018). Although the expression of these genes is important for the efficacy of ICB therapy, their expression as a function of aging has not been studied. In order to understand the relationship of the expression of these genes and age, we performed differential expression analysis in both TCGA and CLS tumor samples, as well as normal GTEx tissue samples.

**Figure 2.**
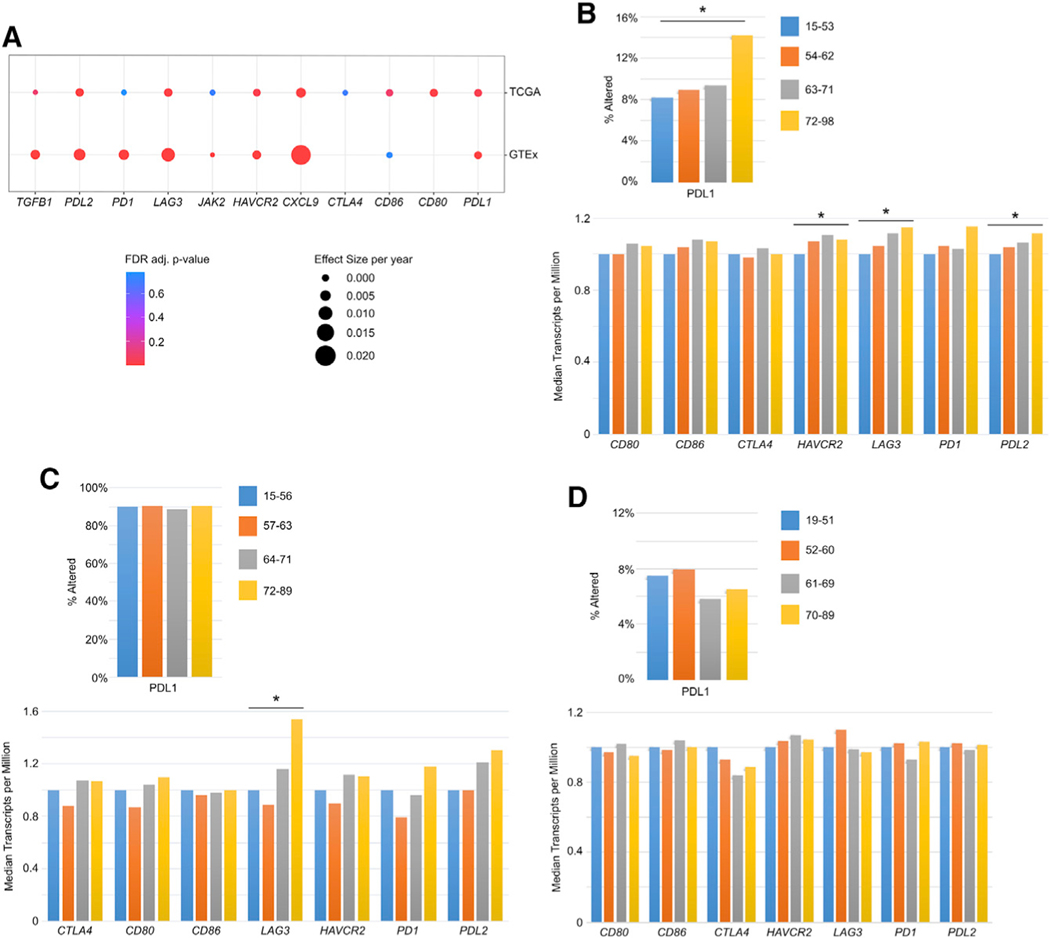
Patient age at diagnosis correlates with increased expression of immune checkpoint genes in some cancer types (A) Dot plot of differential expression statistics for immune checkpoint therapy-related genes with age. Compares results from pan-cancer TCGA samples and pan-tissue GTEx samples. (B) Caris Life Sciences colorectal cancer cohort PDL1 immunohistochemistry (top) and immune checkpoint gene expression data in median transcripts per million (bottom). Asterisk indicates a false discovery rate (FDR)-adjusted *p < 0.05. (C) Caris Life Sciences HPV-negative head and neck cancer cohort PDL1 immunohistochemistry (top) and immune checkpoint gene expression data in median transcripts per million (bottom). Asterisk indicates a FDR-adjusted *p < 0.05. (D) Caris Life Sciences breast cancer cohort PDL1 immunohistochemistry (top) and immune checkpoint gene expression data in median transcripts per million (bottom).

In TCGA, we identify that of these listed genes, *PDL1, CD80, HAVCR2, LAG3, PDL2,* and *CXCL9* expression significantly increase with age (Figure 2A; p values and effect sizes are provided in Table 1), including cancer type as a covariate. We compare these findings with reference non-cancer samples from GTEx to assess whether there is any age-associated expression change in these genes in normal tissues (Figure 2A; Table S1). As in the cancer tissue samples in TCGA, *PDL1, HAVCR2, LAG3, PDL2*, and *TGFB1* expression significantly increase with age among GTEx normal samples pan-tissue, while *JAK2* significantly decreases,andnosignificantchangeisidentifiedinCD86expression (p values and effect sizes are in Table S1). *CTLA4* and *CD80* are very lowly detected across samples in GTEx and therefore do not enable comparison (see STAR Methods). These results indicate that the gene expression differences observed in tumor samples are likely largely the result of the systemic effects of aging, possibly involving the higher levels of inflammation that have been reported in older individuals (Fulop et al., 2018; Kovtonyuk et al., 2016).

We further investigate age-related changes in expression of these genes within each cancer type in CLS, TCGA, and META-BRIC. Analysis of the CLS cohorts of colorectal, head and neck, and breast cancers identifies a significant increase in PDL1 expression via immunohistochemistry (q = 1.03 × 10^−9^), as well as increases in *HAVCR2* (q = 0.0077), *LAG3* (q = 7 × 10^−4^), and *PDL2* (q = 0.0357) RNA expression in colorectal cancer in elderly patients (Figure 2B) and a significant increase in *LAG3* expression (q = 0.0112) in patients with HPV-negative head and neck cancer (Figure 2C), while no significant changes in immune checkpoint gene expression were identified in the breast cancer cohort (Figure 2D). We identify significantly increased expression of *PDL2* and *CXCL9* in lung adenocarcinoma with age in TCGA (Figure S3). Head and neck, colorectal adenocarcinomas, and gastric cancer tumors in TCGA also demonstrate increased age-related expression trends in *PDL1*, although they do not reach statistical significance, while melanoma, breast, bladder, and kidney cancers do not show any age-related association (Figure S3). We note that some TCGA studies have relatively low numbers of patient RNA-seq samples, limiting the statistical power of subtype-specific analyses, particularly when evaluating two highly heterogeneous variables (age and cancer type). Finally, we evaluate differential expression with patient age among METABRIC breast cancer samples and identify a significant decrease in *CD80* expression (q = 0.044) and no significant differences in the other immune-checkpoint-related genes assayed with age (Supplemental Data S2).

### Gene set enrichment indicates age-related signaling changes in pathways associated with ICB response

**Figure 3.**
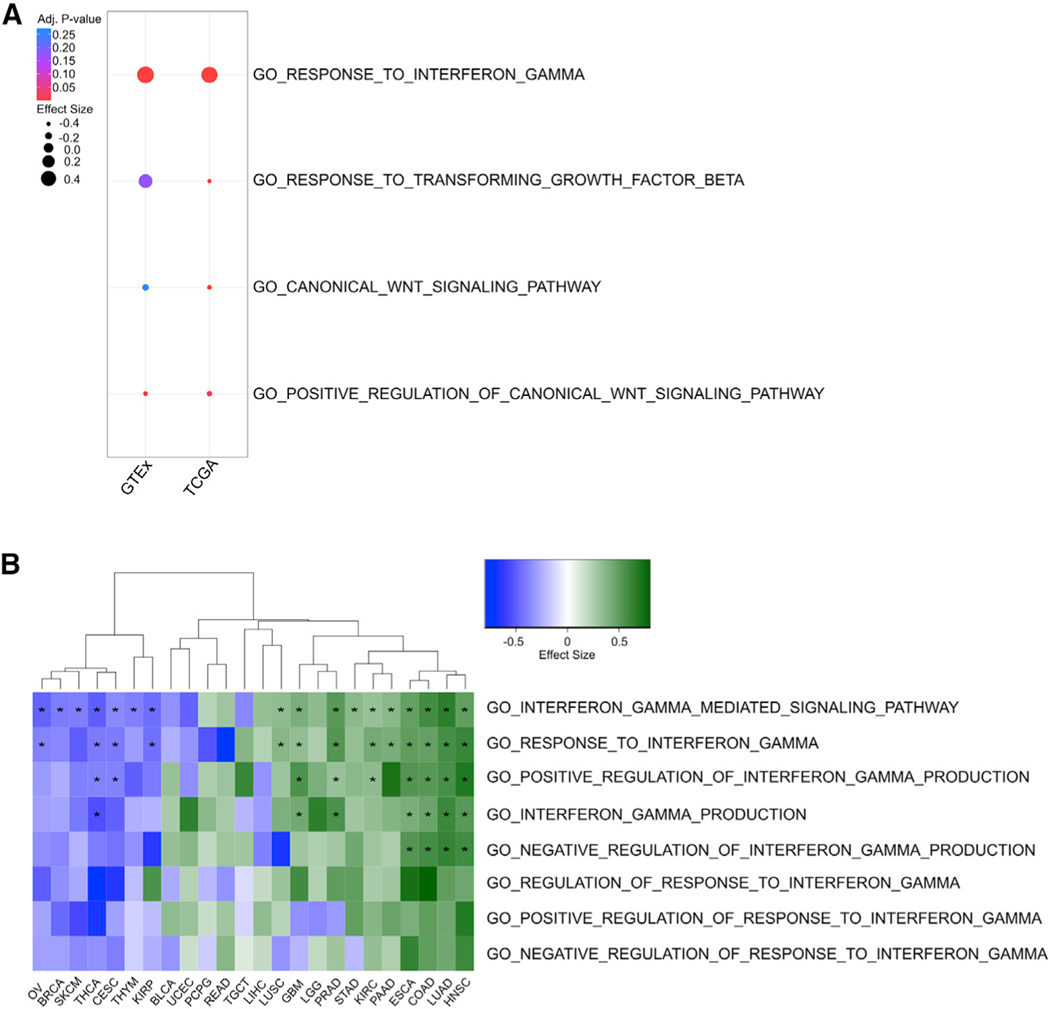
Patient age associates with a more immune-stimulatory signaling tumor microenvironment (A) Dot plot of gene set enrichment results pan cancer in TCGA and pan-tissue in GTEx for interferon gamma, TGF-β, and canonical WNT pathways. (B) Heatmap of estimated effect sizes for gene set enrichment across TCGA studies for all interferon-gamma-related GO terms. Positive values indicate increased enrichment with increasing age. Asterisk indicates a FDR-adjusted *p < 0.05.

To further evaluate the role of transcriptional regulation on ICB biomarkers, we performed additional analysis of several molecular pathways that have been shown to predict patient response to ICB therapies, including high interferon gamma signaling (Higgs et al., 2018), low TGF-β signaling (Tauriello et al., 2018), and low WNT pathway signaling (Martin-Orozco et al., 2019). These pathways are indicative of an immunostimulatory and immune-inhibitory tumor microenvironment, respectively. To determine if the expression of any of these pathways is altered with patient age, we perform differential expression and Gene Ontology (GO) term enrichment on both TCGA tumor samples and GTEx normal samples. We observe increased enrichment of the GO_RESPONSE_TO_INTERFERON_GAMMA term in both TCGA tumors (normalized effect size [NES] = 2.05; q = 1.19 × 10^−3^) and GTEx normal (NES = 2.37; q = 2.84 × 10^−3^) samples with increasing age, decreased GO_RESPONSE_TO_TRANSFORMING_GROWTH_FACTOR_BETA in TCGA tumors (NES = −2.11; q = 1.03 × 10^−3^), decreased signaling through the GO_CANONICAL_WNT_SIGNALING_PATHWAY in TCGA tumors (NES = −2.00; q = 1.03 × 10^−3^), and decreased GO_POSITIVE_REGULATION_OF_CANONICAL_WNT_SIGNALING_PATHWAY in both TCGA tumors (NES = −1.57; q = 0.021) and GTEx normals (NES = −1.69; q = 5.64 × 10^−3^) (Figure 3A).

We further sought to evaluate the impact of aging on these pathways within cancer types. Of particular note, we observe increased enrichment of interferon gamma signaling terms with age in most ICB-approved cancers, including colon, esophageal, head and neck, kidney, lung, and gastric cancer cohorts (Figure 3B). However, in melanoma and breast cancer cohorts, interferon gamma signaling significantly decreases with age, and bladder cancers demonstrate no significant difference (Figure 3B). We identify decreased TGF-β signaling in breast, kidney, and gastric cancers; increased TGF-β signaling in lung and bladder cancers; and no significant change in the other aforementioned cohorts (Figure S4A). We additionally observe decreased WNT signaling terms in breast, esophageal, kidney, melanoma, and gastric cohorts; increased signaling in lung and bladder cancers; and no significant change in colon and head and neck cohorts (Figure S4B). Although these results display heterogeneity in the relationship of age and the expression of tumor immune pathways, they suggest a general shift toward a more immunostimulatory signaling environment in older patients in most ICB-approved cancer types, which would be expected to improve response to ICB therapies. The similar association identified in the normal tissues corresponding to these tumor types from GTEx data further indicates that this shift may relate to the general increase in inflammation that has been repeatedly linked to biological aging (Kovtonyuk et al., 2016; Fulop et al., 2018; Franceschi et al., 2000).

### Age-related changes in promoter methylation align with most of the observed shifts in gene and pathway expression

Due to previous work suggesting that DNA methylation regulates tumor expression of *PDL1* (Asgarova et al., 2018; Micevic et al., 2019), we hypothesize that, to the extent the observed expression increases in immune checkpoint genes occur within individual cancer types, they are driven by changes in DNA methylation. We leverage merged 450k and 27k methylation array data from TCGA (Thorsson et al., 2018) and use Illumina methylation array mappings to annotate CpGs to the promoters of specific genes. We find that of two probes annotated to the *PDL1* promoter region, methylation of one of the probes significantly decreases with age pan-cancer (q = 3.27 × 10^−10^; −0.3% of mean probe intensity per year of age), while the other does not demonstrate any significant change (q = 0.232). Methylation of CpGs annotated to the promoters of *LAG3, CTLA4, CD86, CD80,* and *HAVCR2* also decreases with age pan-cancer (Table 2). One CpG annotated to the TGF-β promoter is hypermethylated with age, while another has no significant change (Table 2). No CpGs within these data were annotated to *CXCL9*. We further investigate CpG methylation within individual cancer types. Similar to the gene expression patterns, we observe considerable heterogeneity in these data across cancer types (Figure S5). Although most cancers approved for ICB therapy have decreasing promoter methylation trends with age among the majority of these CpGs, many do not reach statistical significance. However, both gastric and esophageal cancers demonstrate significant decreases in promoter methylation of *PDL1* and *CD86* with increasing age.

We additionally investigate whether age-related promoter methylation appears in concordance with observed changes in pathway expression pan-cancer in TCGA samples. Although there is no significant change in methylation of gene promoters annotated to the GO_RESPONSE_TO_INTERFERON_GAMMA term (q = 0.663), GO_RESPONSE_TO_TRANSFORMING_GROWTH_FACTOR_BETA promoter methylation increases with age (NES = 1.97; q = 4.36 × 10^−4^), as does GO_CANONICAL_WNT_SIGNALING_PATHWAY (NES = 2.05; q = 4.36 × 10^−4^). These promoter methylation increases are concordant with the observed expression decreases of these pathways with increasing age. Taken together, these results suggest that age-related methylation changes, as have been reported to occur in normal aging and oncogenesis (Easwaran and Baylin, 2019; Easwaran et al., 2012; Horvath, 2013), may drive some of the observed age-related expression-related changes in ICB therapy biomarkers.

### Deconvolution of immune-cell-type abundance in tumor samples reveals an age-related decrease in T cell abundance and increase in macrophage abundance

Ultimately, ICB response relies on the balance between cellular subtypes contributing to immune attack and immunosuppression in the ITME. As a result, the immune cell infiltrate of the tumor microenvironment has been shown to be associated with response to ICB therapies, particularly the relative infiltration of T cells and natural killer (NK) cells with macrophages and myeloid derived supressor cells (Frankel et al., 2017). The large number of primary tumor transcriptional profiles across disease subtypes available from TCGA provides a unique cohort to estimate the impact of age on tumor immune cell composition. We apply the MIXTURE immune-cell-type deconvolution algorithm (Fernández et al., 2021) to infer the absolute proportions of immune cell types from RNA-seq data derived from pan-cancer TCGA samples. The algorithm provides an absolute proportion that describes the portion of total immune content that a particular immune cell type makes up in a sample but is normalized to be comparable across all samples in the dataset by multiplying the inferred relative proportion by a scaling factor that measures the total immune content in the sample. We then fit a linear model with age, including cancer type and patient sex as covariates, for each immune cell type (listed in Table S2) to assess changes in immune cell infiltration as patients age. We find that overall T cell abundance slightly but significantly decreases with age in the ITME (−6.03 × 10^−4^ per year; mean proportion, 0.198; q = 0.00175) (Figure S6A; Table 3), while macrophages slightly but significantly increase in abundance (1.08 × 10^−3^ per year; mean proportion, 0.662; q = 4.45 × 10^−4^) (Figure S6B; Table 3). Detectable changes in the infiltration of NK cells, dendritic cells, B cells, and other myeloid populations do not occur with age pan-cancer (Table 3).

To compare the effect of aging in the ITME with that on the immune cell compositions of normal tissues, we applied MIXTURE to GTEx consortium RNA-seq data of post-mortem samples from individuals without cancer (GTEx Consortium, 2017) to infer cell-type abundance across tissues. These results provide a non-cancer baseline for immune changes that occur across many individuals of varying ages to compare with our observations from tumor data. Similar to our TCGA and METABRIC analyses, we fit a linear model to each cell type in order to determine associations between cell-type abundance and age both across and within normal tissues. In contrast with our findings in the pan-cancer ITME, in pan-tissue analyses, we observe a significant increase in overall T cell absolute proportion with age (8.97 × 10^−4^ per year; mean proportion, 0.106; q = 0.001) (Figure S7A; Table 3). We further fail to find significant changes in macrophage levels (q = 0.870) with age (Figure S7B). Additionally, we observe increases in NK cell proportion (0.0019 per year; mean proportion, 0.062; q = 5.13 × 10^−14^) (Figure S7C) and decreases in other myeloid cell (monocytes, mast cells, eosinophils, neutrophils) (−0.0018 per year; mean proportion, 0.470; q = 0.0235) (Figure S7D) proportion that were not found among TCGA tumor samples. Recall that each effect size must be evaluated relative to the average proportion of immune infiltrate that cell type makes up (e.g., NK cells are expected to increase in abundance 153% over 50 years of life on average, while myeloid cells are expected to decrease only 19% over that same period despite essentially the same absolute proportion change per year). These results indicate differences between systemic immune aging and the effects of age on immune tumor infiltrate. Most notably, a very large systemic increase in NK cell abundance does not appear to be reflected in the tumors of older patients.

**Figure 4.**
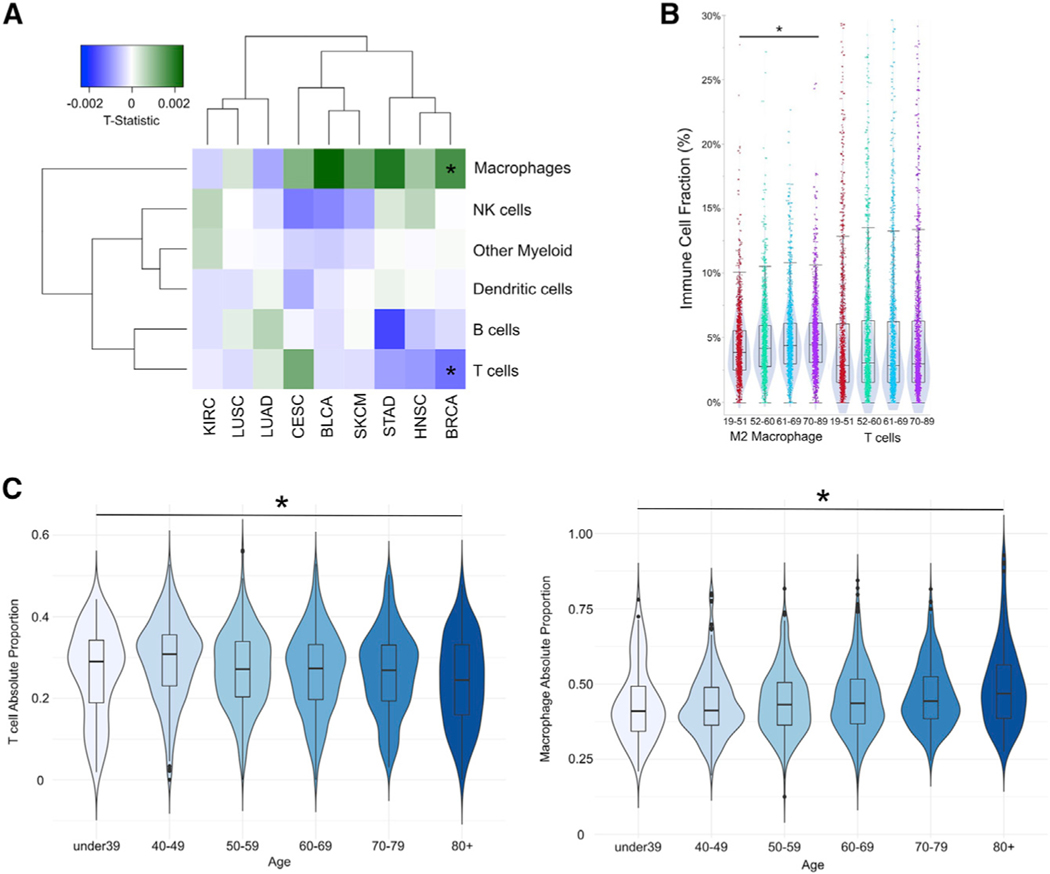
Macrophage infiltration increases with age in patients with breast cancer (A) Heatmap displaying the effect size coefficient estimates from linear models fit between immune-cell-type absolute proportion and patient age in each TCGA cancer type study. Green squares represent an increase in abundance of that immune cell type with increasing age, white represents no change, and blue a decrease. Asterisk indicates a FDR-adjusted *p < 0.05. (B) Violin plots from the Caris Life Sciences breast-cancer cohort (n = 6,462) corresponding to tumor-infiltrating immune cell fraction across different age groups among M2 macrophages and T cells. Asterisk indicates a FDR-adjusted *p < 0.05. (C) Violin plots from 1,818 patients with breast cancer from METABRIC, comparing T cell and macrophage absolute proportion across patient age groups. Asterisk indicates a FDR-adjusted *p < 0.05.

To determine the variance in age-related effects that occur within different cancer types, we then evaluate the association between age and immune composition for each cancer type with at least 100 samples that could be successfully deconvoluted by the MIXTURE algorithm. Non-significant deconvolution is generally due to a low content of the immune cells the algorithm searches for, and with this filtering only 8 tumor types in TCGA have over 100 samples after filtering. Although several cancer types demonstrate age-related trends in T cell and macrophage abundance, these are found to be statistically significant only in breast cancers (Figure 4A). To determine if these results are robust across cohorts and whether lack of statistical significance in some cancer types is related to a lack of statistical power, we further examine large breast, head and neck, and colon cancer cohorts produced by CLS. As methodological validation, a different immune-cell-type deconvolution program, quanTIseq (Finotello et al., 2019), was used to estimate cell-type abundance from RNA-seq data. Among 6,462 patients with breast cancer, a significant increase in M2 macrophage infiltration was identified with increasing age, but no significant difference was observed among infiltrating T cell abundance (Figure 4B). In contrast, within 7,924 patients with colorectal cancer and 527 patients with HPV-negative head and neck cancer, no significant differences in macrophage or T cell immune cell fraction are observed (Figure S8) (full results of immune infiltration and age are reported as Supplemental Data S3). We further investigate this association among 1,818 METABRIC patients with breast cancer, again using MIXTURE for immune-cell-type deconvolution. We identify a similar decrease in T cell abundance with age (−6.57 × 10^−4^ per year; mean proportion, 0.268; q = 0.00188) and increase in macrophage abundance with age (1.38 × 10^−3^ per year; mean proportion, 0.45; q = 8.21 × 10^−9^) (Figure 4C), as well as a significant decrease in B cell abundance (4.22 × 10^−4^ per year; mean proportion, 0.0554; q = 8.51 × 10^−4^) that we did not observe in TCGA breast cancer data. This analysis thus identifies age-related macrophage proportion increases with patient age across three different breast cancer cohorts (TCGA, METABRIC, and CLS) using two different computational microdissection methods.

### Patient age associates with little to no detectable difference in survival outcomes after ICB treatment

Although the genomics datasets we have examined can help uncover the molecular and cellular pathways of mechanistic biomarkers for ICB that are altered by age, they cannot directly evaluate therapeutic response. Previous analyses of the impact of age on ICB therapeutic efficacy in clinical trials (Kugel et al., 2018; Elias et al., 2018; Daste et al., 2017; Jain et al., 2020) have remarked on the limited numbers of older patients treated with ICB available for their analyses and the need for further investigation of this subject. To provide additional insight into this question, we investigate the relationship between age and outcome. A recently published cohort of anti-PD1-treated patients with renal cell carcinoma (Braun et al., 2020) had age available for 985 patients, along with progression-free survival (PFS) and overall survival (OS). We identify no statistically significant difference in PFS or OS with age, both based on a log rank test (p = 0.25 and p = 0.29, respectively) (Figure S9) and multivariate Cox proportional hazards analysis (hazard ratio [HR] = 0.994 [0.987–1.001], p = 0.09 and HR = 1.001 [0.994–1.009], p = 0.72, respectively), including sex, number of prior therapies, and metastatic origin as covariates. We also investigate survival differences in 11,888 ICB-treated patients with melanoma, lung, kidney, head and neck, or urothelial cancers collected by the US Department of Veterans Affairs (USVA) (La et al., 2020). A multivariate Cox proportional hazards model fit for patient OS, including cancer type and sex as covariates, identifies a statistically significant reduction in OS, of small effect size, for patients with increasing age (HR = 1.005 [1.001–1.009]; p = 0.01).

### High-throughput molecular databases inform an atlas of immune aging in cancer and healthy tissues

Understanding the impact of patient age on likelihood of response to immunotherapies is a subject of clear clinical relevance, and investigating relevant biomarkers of said response forms the central focus of this work. Still, the comprehensive analysis of these data was based on general characterization of aging-related molecular shifts in tumors and the tumor microenvironment. To that end, we provide a web application containing these results to enable custom analyses of the relationship of age to molecular changes genome-wide: http://www.lab-apps.onc.jhmi.edu/CAMAAtlas. The CAMA atlas is informed from analysis of 9,523 patients across 31 cancer types from TCGA, 37,961 patients across 8 cancer types from GENIE, 1,818 patients with breast cancer from METABRIC, and a pan-tissue reference of 948 non-cancer individuals from GTEx.

In brief, the web-based application includes distinct panels for each of the analyses of distinct molecular modalities and datasets, based upon the data that are available from each cohort. The application allows for exploration of associations of TMB with age by cancer subtype in both TCGA and GENIE. The application further allows for customized evaluation of tumor-subtype changes relative to tissue-specific changes in gene expression through differential expression analyses in TCGA and GTEx, respectively. Although the analyses presented in this study are limited to gene expression changes in ICB biomarkers, the CAMA web application allows users to search for genes of interest across the entire genome allowing for evaluation of further age-related changes in the immune context and beyond. The application allows for further evaluation of the regulatory changes associated with these transcriptional alterations through GO enrichment analysis (TCGA and GTEx) and DNA methylation changes with patient age (TCGA). The CAMA atlas is thus intended to act as an initial resource for further studies of the relationship between molecular features of cancers and aging. The relationship of a particular molecular feature (gene expression, gene promoter methylation, pathway enrichment, cell-type abundance) with age can be queried by individual cancer type or across cancers. This atlas is meant to provide a resource that broadly characterizes cancer genomic associations with patient age and can be used to perform customized analyses. These relationships are often available in multiple cohorts, allowing for computational validation of identified associations.

## DISCUSSION

This study presents an atlas of age-related shifts in the genomic, transcriptomic, and immune tumor environment. The effect of patient age on tumor characteristics has not been thoroughly explored in most cancer types. Here we analyze genomics and clinical databases from a total of 77,732 cancer patients with 31 different cancer types to generally characterize relevant associations between age and these molecular markers, which we provide the broad results of as the CAMA atlas (http://www.lab-apps.onc.jhmi.edu/CAMAAtlas).

We hypothesize that the relationship between age and cancer makes understanding the impact of aging on cellular and molecular pathways an important consideration for precision medicine. Indeed, the general link between increased age and reduced immune effectiveness has naturally inspired caution and concern about the treatment of elderly patients with ICB therapies. Therefore, in this study, we leverage multiple large-scale cancer genomic cohorts to characterize the impact of age on established ICB biomarkers and contextualize previous clinical findings that older patients counterintuitively experience either no reduced benefit or increased benefit from ICB immunotherapies as compared with younger patients (Kugel et al., 2018; Elias et al., 2018; Jain et al., 2020). Our analysis identifies several possible explanations for these data based on currently established and developing predictors of ICB response. Patient age at diagnosis is associated with increases among several biomarkers associated with effective ICB response, including notably increased TMB, increased expression and decreased promoter methylation of immune checkpoint genes, increased interferon gamma signaling, decreased TGF-β signaling, and decreased canonical WNT signaling. The induction of these immunostimulatory biomarkers may be related to normal mutational accumulation with age, the increased inflammation that has been observed in normal systemic aging (Kovtonyuk et al., 2016; Fulop et al., 2018; Franceschi et al., 2000), and previously identified age-related methylation changes (Easwaran and Baylin, 2019;Easwaran et al., 2012; Horvath, 2013). Expected to act in opposition to these immune effects, we observe concurrent features of immunosuppression with age, such as decreased TCR diversity and T cell infiltration, as well as increased macrophage abundance, in some cancer types. However, it is critical to note that the effect size of TCR decreases with age in pan-cancer is quite small (on average, a −0.26% change per year of age). Further, the decrease in T cell abundance and increase in macrophage abundance is not only small (on average, −0.3% and 0.16% change per year, respectively), it is also only statistically significant in one individual cancer type: breast. Altogether, these results support an adapting immune landscape with age that nonetheless retains characteristics associated with effective ICB response. Nonetheless, we note that all results of this work are correlative, and thus a large-scale prospective study collecting genomics for immunotherapy-treated elderly patients is warranted to generate a causal understanding of the effects of age on the immune response to cancer.

We complement our molecular studies with corresponding analysis of patient outcomes from large-scale clinical databases for two large cohorts of ICB-treated patients containing patients across an array of ages. Among the renal cell carcinoma cohort published by Braun et al. (2020), we identify no significant difference in PFS or OS with age, supporting the results of previous clinical studies. However, among a large group of patients collected by the USVA, we find a small decrease in OS with age. It is notable that this slight OS difference observed could be related to general age-related frailty rather than differences in immunological efficacy. This point is supported by previous work published on this USVA cohort, which showed that a frailty status assessment considerably better differentiated therapeutic response in each cancer type than did patient age (La et al., 2020). The immunological biomarkers assessed in this study further support the interpretation that most of the small worsening in survival outcomes sometimes observed for older patients is the result of increased systemic frailty rather than decreased efficacy of the therapy itself. Future large-scale cohort studies of aged populations with combined outcomes, frailty measures, and genomics data are critical to fully delineate the relative impact of frailty and functional mechanisms of ICB response on its efficacy in the elderly population.

This study additionally includes normal tissues in our analyses in order to understand whether the associations noted appear to be a normal consequence of age or an interaction between aging and tumor biology. The associations established between age and ICB biomarkers largely recapitulate in GTEx normal samples (ICB gene expression, immune pathway enrichment), or, when not assessed here, have already been thoroughly established in the literature (TCR diversity [Aspinall and Andrew, 2000; Yager et al., 2008; Britanova et al., 2014; Egorov et al., 2018]; mutational accumulation [Morley, 1998]). The major exception identified to this concordance between tumor and normal aging is the large increase in NK cells with age in normal tissues, which has been previously identified in the literature (Solana and Mariani, 2000; Gounder et al., 2018). This NK cell increase is not observed pan-cancer and is observed in only one cancer type cohort studied, Caris breast, where there was only a ∼5% increase, on average, between the youngest and the oldest patients, compared with a ∼150% increase in GTEx samples. This result suggests that although NK cell proportion increases with age, they either are not able to proportionately respond to immune stimuli and infiltrate into the aged tumor tissues or that aging biology interacts with tumor biology to inhibit the infiltration of NK cells. NK cells have been shown to play a significant role in ICB efficacy and general tumor immunity (Shimasaki et al., 2020; Freeman et al., 2019; Lo et al., 2020; Jhunjhunwala et al., 2021), and thus this observation may be therapeutically relevant, particularly if these accumulated NK cells can be stimulated to infiltrate the tumors of elderly patients.

Beyond their relevance to ICB alone, the molecular and cellular changes inferred from the CAMA atlas may support selection of precision medicine strategies based on molecular and cellular changes in elderly patients. For example, we identify macrophage increases with age in three different breast cancer cohorts (TCGA-BRCA, METABRIC, CLS-Breast) with two different computational microdissection methods (MIXTURE and quanTIseq). Combination therapeutics to target immunosuppressive cells are emerging as a common therapeutic approach to sensitize tumors to immunotherapies. For example, there are several strategies currently in development to target tumor-associated macrophages (Chanmee et al., 2014; Lee et al., 2019; Poh and Ernst, 2018). These results suggest that elderly patients with breast cancer may be particularly promising candidates for these therapies. Thus, characterizing age-related changes in these distinct cellular populations in the tumor microenvironment can further illuminate combination therapeutic strategies specific for elderly patients.

To ensure that our data were sufficiently powered to analyze aging-related effects of tumors and their microenvironments, we leverage large-scale databases that contain predominantly bulk profiling technologies. It is important to note the limitations of bulk expression data for some of the analyses in this work. Notably, our aging-related analyses of cell types relies on computational microdissection to provide estimates of proportional representation on each cell type in each sample studied. However, these techniques are effective only for samples with substantial immune infiltration, limiting the number of tumors that could be included in this analysis. Moreover, these bulk data do not enable discovery of cell-type-specific molecular pathways that are altered by aging. Some computational methods have been developed to attempt to regress out effects of individual cell types on bulk expression data to perform such cell-type-specific differential expression analysis. However, these techniques will be confounded in cases in which immune genes also serve as cell-type markers, limiting the applicability of these techniques for the analyses in our atlas. A further limitation of computational microdissection methods used is that they estimate cell-type abundance, but not cell state. Single-cell data are essential to further evaluate immune cell functionality and quality in the ITME. Although large-scale single-cell studies of aging have been generated in healthy tissue for mouse models (Tabula Muris Consortium, 2020), to date these studies are for small cohorts in tumors that are not sufficiently powered to identify immune cell state transitions associated with aging. Therefore, future single-cell pan-cancer characterization from projects such as the Human Tumor Atlas Network (Rozenblatt-Rosen et al., 2020) will be critical to validate these results and further expand our atlas to delineate the role that aging-related changes to immune cell function play in cancer.

## STAR★METHODS

### RESOURCE AVAILABILITY

#### Lead contact

Queries regarding this work should be directed to Elana J. Fertig (ejfertig@jhmi.edu).

#### Materials availability

This study did not generate new unique reagents.

#### Data and code availability

- This paper analyzes existing, publicly available data. These accession numbers for the datasets are listed in the Key resources table.
- All analysis code is available on GitHub at: https://github.com/rossinerbe/ImmuneAgingAnalysis, the repository for which is archived with Zenodo at https://doi.org/10.5281/zenodo.5119645.
- Any additional information required to reanalyze the data reported in this paper is available from the lead contact upon request.

### EXPERIMENTAL MODEL AND SUBJECT DETAILS

Relevant information on the publicly available cohorts analyzed in this work are provided as supplemental data. Further information on these cohorts is available at the sites list in the Key resources table.

### METHOD DETAILS

#### RNA-Sequencing Data

TCGA RNA-sequencing data processed and normalized according to https://docs.gdc.cancer.gov/Data/Bioinformatics_Pipelines/Expression_mRNA_Pipeline/ was downloaded from the GDC Data Portal on August 8th, 2019, filtering for all TCGA samples with patients above 30 years of age. Patients under 30 were excluded to focus on ITME changes in adult populations, which are more likely to generalize to the majority of cancer patients.

GTEx RNA-sequencing counts version 8 were downloaded from the GTEx Portal on November 12th, 2019. Only individuals over 30 were included in the final analysis, to be comparable with filtering of TCGA. Characteristics of these cohorts are listed in Table S4.

METABRIC RNA-seq counts were downloaded from cBioPortal on October 20th, 2020 as provided by (Pereira et al., 2016).

#### TMB Data

To find the association of patient age and number of tumor mutations we downloaded the mutation counts provided for each sample pan-cancer in TCGA from the GDC data portal on August 8th, 2019. GENIE TMB counts were downloaded from the GDC data portal for all patients with cancers on January 13th, 2021.

#### TCR Clonality Data

TCR clonality was estimated from TCGA RNA-seq data using the miTCR algorithm (Bolotin et al., 2013), as previously published by (Thorsson et al., 2018). These data were published publicly on the GDC data portal and downloaded from the link provided in the Key resources table for use in this study.

#### DNA Methylation Data

Merged 450k and 27k DNA methylation array data preprocessed by Thorsson et al. (2018) were published on the GDC data portal and downloaded from the link provided in the Key resources table for use in this study.

### QUANTIFICATION AND STATISTICAL ANALYSIS

All statistical analyses were performed using R version 4.0.2. Statistical significance is evaluated as p < 0.05 after the Bonferroni-Hochberg procedure was applied in cases of multiple hypothesis testing.

#### Modeling the Age Associations of Number of Tumor Mutations and Normalized TCR Clonality

We transformed the TMB counts data with a natural log, which we used to fit a log linear model and Cox proportional hazards model, using cancer type as a covariate in the log linear model and cancer type and age at diagnosis as covariates for the Cox model. We additionally fit log linear models between TMB and patient diagnosis age within each TCGA cancer type study.

TCR clonality is assessed using miTCR (Bolotin et al., 2013) results previously published by Thorsson et al., 2018 (Thorsson et al., 2018). Our immune cell type deconvolution results demonstrate there may be decreased infiltration of T cells with increasing age, so to avoid biasing our results, the Shannon Entropy is multiplied by the number of unique TCR clones divided by the total number of TCR reads. We then fit a linear model for the association of age with this TCR clonality measure, including patient sex and cancer type as covariates. We again use a Cox Proportional hazards model to assess if normalized Shannon entropy is a relevant survival prognostic, using the same survival function and covariates as described above. We additionally fit linear models between normalized Shannon entropy and patient diagnosis age within each TCGA cancer type study.

#### Differential Expression Analysis with Age

Differential expression analyses from both TCGA and GTEx data were performed on all samples from individuals of at least 30 years of age. The R edgeR package version 3.30.3 was used for normalization and identification of differentially expressed genes with age. Age at diagnosis was modeled as a continuous variable, including cancer type as a covariate for the TCGA analysis and tissue type as a covariate for the GTEx analysis. Immune cell type proportions were included as covariates in each analysis to account for age-related differences in abundance. Genes were considered differentially expressed below an FDR adjusted p value of 0.05. Differential expression analysis for diagnosis age was analogously performed on each cancer type separately that had at least 100 samples, though cancer type was naturally no longer included as a covariate.

#### Gene Set Enrichment Analysis

The fgsea R package version 1.14.0 (Sergushichev, 2016) was used to perform gene set enrichment analysis from differential expression results with age from TCGA and GTEx, produced as described above. GO terms were downloaded from MsigDB (Liberzon et al., 2011) using the msigdbr R package Version 7.2.1. GO enrichment was determined for all terms both pan-cancer and within each TCGA cancer type study and terms related to Interferon Gamma, TGFb, and WNT were visualized.

#### Differential Methylation Analysis with Age

Merged 450k and 27k DNA methylation array data was used to examine the relationship between age and DNA mathylation. A linear model for diagnosis age was fit using R version 4.0.2 to data from each CpG, including cancer type as a covariate. CpG methylation was considered significantly different with age if the FDR adjusted p value for the diagnosis age term was less than 0.05. Annotations of CpG sites to gene promoters were retrieved from the IlluminaHumanMethylation27k.db R package Version 1.4.8. The same process was repeated among each TCGA cancer type study, using a linear model between each CpG and patient diagnosis age. Gene set enrichment analysis was performed by using the differentially methylated CpGs that are annotated to gene promoters. This analysis was performed as described above using the R fgsea package version 1.14.0 (Sergushichev, 2016).

#### Immune Cell Type Deconvolution from Bulk RNA-Sequencing Data

The MIXTURE algorithm (Fernández et al., 2021) builds on the nu-Support Vector Regression framework used by CIBERSORT (Newman et al., 2015) for particular use with noisy tumor samples. MIXTURE applies Recursive Feature Selection to make the cell type deconvolution more robust to noise and collinearity, and was thus designed to improve performance on tumor data.

We run MIXTURE using a population-based null distribution and the nu-SVM Robust RFE method on the preprocessed RNA-sequencing data from both TCGA and GTEx. A signature expression matrix (LM22 from Newman et al.) (Newman et al., 2015) is used to determine the proportion of 22 immune cell types in each sample. MIXTURE returns both relative and absolute proportions of immune cells. Absolute proportions were used for all analyses of TCGA and GTEx datasets. MIXTURE provides a p value for the cell type deconvolution performed. Only samples with a deconvolution p value less than 0.05 were used in the final analyses, leaving 3576 patient samples remaining in TCGA and 1689 in GTEx. A further 29 TCGA patients had received treatment prior to sample collection, and were removed to avoid biasing of results.

#### Modeling the Association of Immune Cell Type with Age

Linear models are fit to investigate the association between the absolute proportion of each immune cell type and the initial diagnosis age in TCGA. The models are fit separately for each cancer type as well as jointly with cancer type and patient sex as covariates. Significance is assessed using Benjamini-Hochberg FDR correction for multiple testing across all cell types tested.

Higher order cell types are defined by adding together individual substituent cell type values and dividing by the sum of all cell types, the result of which is used as the predictor variable in the linear model (which immune subtypes correspond to which higher order cell types is shown in Table S1).

GTEx data was similarly analyzed using linear models, including sex and tissue type as covariates.

#### Survival Modeling for Patient Age among Braun et al. (2020) and USVA cohorts

We fit multivariate Cox proportional hazards models to survival data from 985 anti-PD1 treated patients with renal cell carcinoma collected by Braun et al. (2020) (using progression free survival and overall survival data provide in table S1 of Braun et al., 2020) and to 11,888 ICB treated patients with melanoma, lung, kidney, head and neck, or urothelial cancers, collected by the United States Department of Veteran Affairs. This model was fit using the R survival package version 3.1–12. We additionally produce Kaplan-Meier survival curves based on the Braun et al., 2020 dataset, separating the curves into 65 and under and 66 and older age groups for each dataset. These curves were fit using the R survival package version 3.1–12 and the R survminer package version 0.4.8.

#### Caris Life Sciences Data and Analyses

15,557 Caris samples were analyzed using next-generation sequencing (NextSeq, 592 Genes and WES, NovaSEQ), IHC and WTS (NovaSeq) (Caris Life Sciences, Phoenix, AZ). PD-L1 expression was tested by IHC using 28–8 and 22c3 (Agilent) and SP-142 (Spring Biosciences) (positive cut-off > 1% for CRC and HNC, > 5% for BC). TMB was measured by totaling somatic mutations per tumor. Immune checkpoint gene expression was normalized to the median expression in the lowest age quartile. Immune cell fraction was calculated by quanTIseq (Finotello et al., 2019). Immunotherapy biomarkers, immune checkpoint gene expression and immune cell fraction was compared across four age quartiles. Median transcripts per million (TPM) were normalized to the median TPM value in quartile 1. Statistical significance was determined using chi-square and Wilcoxon rank sum test and adjusted for multiple comparisons using the Benjamini-Hochberg procedure.

### ADDITIONAL RESOURCES

The CAMA atlas, containing associations of patient age to various molecular features of their tumors: http://www.lab-apps.onc.jhmi.edu/CAMAAtlas.

## Supplementary Material

1

2

3

4

5

## Figures and Tables

**Table 1.** Differential expression of immune checkpoint genes by age in TCGA

Gene	LogFC (per year)	t-Statistic	p value	q value
*CXCL9*	0.007	3.945	0.001	0.003
*PDL2*	0.004	3.380	0.001	0.004
*LAGS*	0.004	3.187	0.001	0.007
*CDSG*	0.004	3.002	0.003	0.012
*HAVCR2*	0.003	2.642	0.008	0.028
*PDL1*	0.003	2.612	0.009	0.030
*CDS6*	0.002	2.131	0.033	0.083
*TGFB1*	−0.002	−2.070	0.039	0.094
*CTLA4*	−0.001	−0.575	0.566	0.693
*JAK2*	0.000	−0.563	0.574	0.700
*PD1*	−0.001	−0.421	0.674	0.779

Differential expression results for immune checkpoint genes and immune-checkpoint-related genes pan-cancer in TCGA. The results are shown for the association with patient diagnosis age, including cancer type as a covariate. LogFC, log fold change for each year of age.

**Table 2.** Promoter methylation of ICB-related genes by age in TCGA

CpG	Gene	Estimate (per year)	t-Statistic	p value	q value	CpG island?
cg01107031	*TGFB1*	0.000218	3.326	0.000881	0.00303	yes
cg16883145	*TGFB1*	−2.73 × 10^−5^	−0.644	0.519	0.662	yes
cg04387658	*CD86*	−0.000984	−7.101	1.31 × 10^−12^	2.39 × 10^−11^	yes
cg08460026	*CTLA4*	−0.000638	−3.888	0.000101	0.000433	no
cg17484237	*HAVCR2*	−0.000810	−5.845	5.19 × 10^−9^	5.10 × 10^−8^	no
cg21572897	*CD80*	−0.000526	−4.385	1.17 × 10^−5^	6.01 × 10^−5^	no
cg26956535	*LAG3*	−0.000114	−2.468	0.0135	0.0343	no
cg01820374	*LAG3*	−0.000410	−4.219	2.47 × 10^−5^	0.000119	no
cg02823866	*CD274*	−2.08 × 10^−5^	−1.512	0.130	0.232	yes
cg19724470	*CD274*	−0.000894	−6.701	2.17 × 10^−11^	3.27 × 10^−10^	no

Table of the linear relationships between methylation of all CpGs annotated to ICB-related gene promoters to patient diagnosis age in TCGA data. Results are pan-cancer from a multivariate linear model that included cancer type as a covariate. Note that effect size estimates are per year of age at diagnosis.

**Table 3.** Immune-cell-type proportion by age in TCGA

	Estimate (per year)	t-Statistic	p value	q value
T cells	−0.0006	−3.44233	0.000585	0.001754
Macrophages	0.001075	3.967909	7.42 × 10^−5^	0.000445
B cells	−0.00028	−1.87737	0.060566	0.121131
NK cells	3.82 × 10^−6^	0.055398	0.955826	0.955826
Dendritic cells	−0.00015	−1.49017	0.136286	0.204429
Misc. myeloid	−4.97 × 10^−5^	−0.50055	0.616722	0.740066

Coefficients, statistics, p values, and q values for the diagnosis age term in the linear model fit for each immune cell type in TCGA data pan-cancer. Cancer type and sex were included as covariates for each of these models. Note that estimated coefficients are per additional year of age at diagnosis. Misc., miscellaneous.

**Table:** KEY RESOURCES TABLE

REAGENT or RESOURCE	SOURCE	IDENTIFIER
Deposited data		

The Cancer Genome Atlas	https://portal.gdc.cancer.gov/	https://figshare.com/articles/dataset/TCGARNA-seq/12030318
Genomics Evidence Neoplasia Information Exchange	https://portal.gdc.cancer.gov/	https://github.com/rossinerbe/ImmuneAgingAnalysis/blob/master/Data/genie_public_clinical_data.tsv
Molecular Taxonomy of Breast Cancer	https://www.cbioportal.org/study/summary?id=brca_metabric	https://cbioportal-datahub.s3.amazonaws.com/brca_metabric.tar.gz
Genotype-Tissue Expression Project	https://www.gtexportal.org/home/datasets	GTEx_Analysis_2017-06-05_v8_RNASeQCv1.1.9_gene_tpm.gct.gz
GDC - The Immune Landscape of Cancer – TCR Statistics	https://gdc.cancer.gov/about-data/publications/panimmune	mitcr_sampleStatistics_20160714.tsv
GDC - The Immune Landscape of Cancer – DNA Methylation	https://gdc.cancer.gov/about-data/publications/panimmune	jhu-usc.edu_PANCAN_merged_HumanMethylation27_HumanMethylation450.betaValue_whitelisted.tsv
Analysis of relationship of patient age and biomarkers of immune response	This manuscript	https://github.com/rossinerbe/ImmuneAgingAnalysis/tree/master/Data

Software and algorithms		

R version 4.0.2	https://cran.r-project.org/bin/windows/base/old/4.0.2/	N/A
edgeR version 3.30.3	https://bioconductor.org/packages/release/bioc/html/edgeR.html	N/A
fgsea version 1.14.0	https://bioconductor.org/packages/release/bioc/html/fgsea.html	N/A
MIXTURE	https://github.com/elmerfer/MIXTURE	N/A
Survival version 3.1–12	https://cran.r-project.org/web/packages/survival/index.html	N/A
quanTIseq	https://icbi.i-med.ac.at/software/quantiseq/doc/	N/A
Analysis code from this manuscript	This manuscript	https://github.com/rossinerbe/ImmuneAgingAnalysis

Other		

IlluminaHumanMethylation27k.db version 1.4.8	https://bioconductor.org/packages/release/data/annotation/html/IlluminaHumanMethylation27k.db.html	N/A

## Highlights

- We investigate the relationship of patient age and ICB therapy biomarkers
- Favorable ICB biomarkers are generally more prevalent in elderly patients
- The CAMA web application provides a multi-omics atlas of aging in cancer
