# Supplementary material for: Evaluating the impact of age on immune checkpoint therapy biomarkers
Source: Cell Rep. Author manuscript; Available in PMC 2022 Jan 13. (PMC8757482; doi:10.1016/j.celrep.2021.109599)
Supplement: 1 [file NIHMS1735652-supplement-1.pdf]

Cell Reports, Volume *36*

Supplemental information

Evaluating the impact of age

on immune checkpoint therapy biomarkers

Rossin Erbe, Zheyu Wang, Sharon Wu, Joanne Xiu, Neeha Zaidi, Jennifer La, David Tuck, Nathanael Fillmore, Nicolas A. Giraldo, Michael Topper, Stephen Baylin, Marc Lippman, Claudine Isaacs, Reva Basho, Ilya Serebriiskii, Heinz-Josef Lenz, Igor Astsaturov, John Marshall, Josephine Taverna, Jerry Lee, Elizabeth M. Jaffee, Evanthia T. Roussos Torres, Ashani Weeraratna, Hariharan Easwaran, and Elana J. Fertig

Supplemental Figure 1 - Tumor mutational burden increases with age in the CLS colorectal and breast cancer cohorts

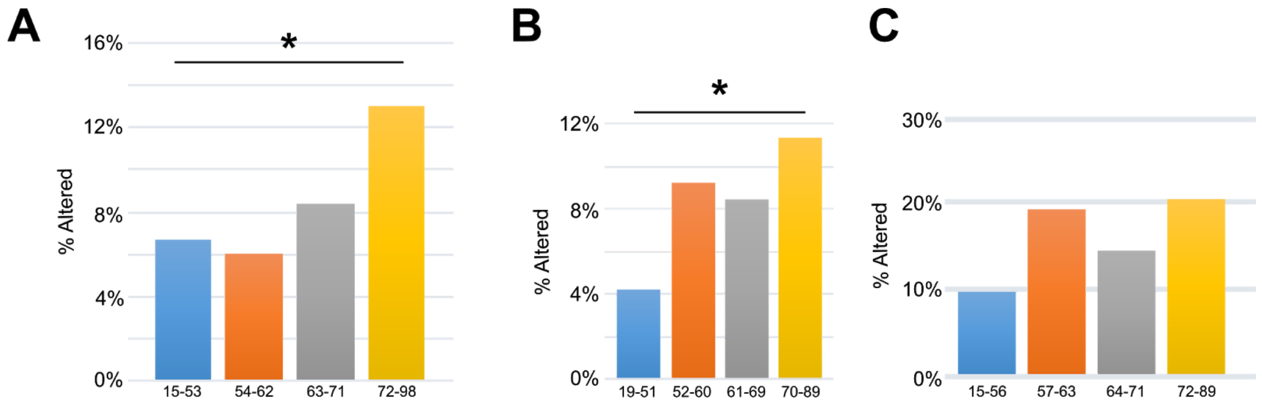

Caris Life Sciences TMB by age-group, related to Figure 1. **A** Bar graph of average percent altered tumor mutational burden counts among CLS colorectal cancer patients. **B** Bar graph of average percent altered tumor mutational burden counts among CLS breast cancer patients. **C** Bar graph of average percent altered tumor mutational burden counts among CLS HPV-negative head and neck cancer patients. * indicates an adjusted p-value less than 0.05.

Supplemental Figure 2 - TCR clonality undergoes changes with patient age in multiple cancer types

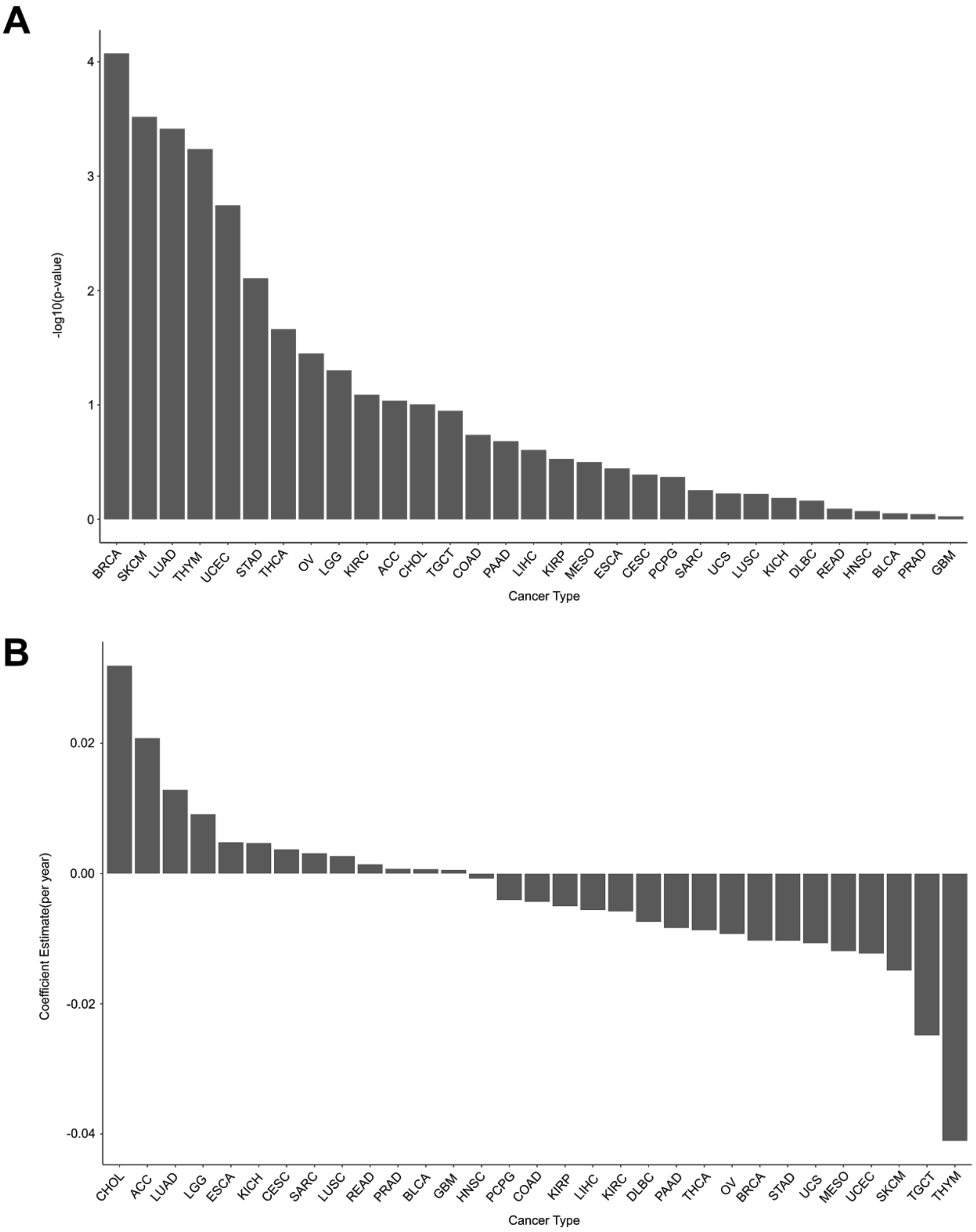

A Barplot of negative log10 p-values for the age term from a linear model with normalized shannon entropy of TCRs across TCGA cancer types. **B** Barplot of coefficient estimates per year of changes in normalized Shannon entropy of TCRs from a linear model fit between patient age and normalized Shannon entropy. Positive values indicate increases with increasing age.

Relates to Figure 1.

Supplemental Figure 3 - Differential expression of immune checkpoint genes with age in TCGA

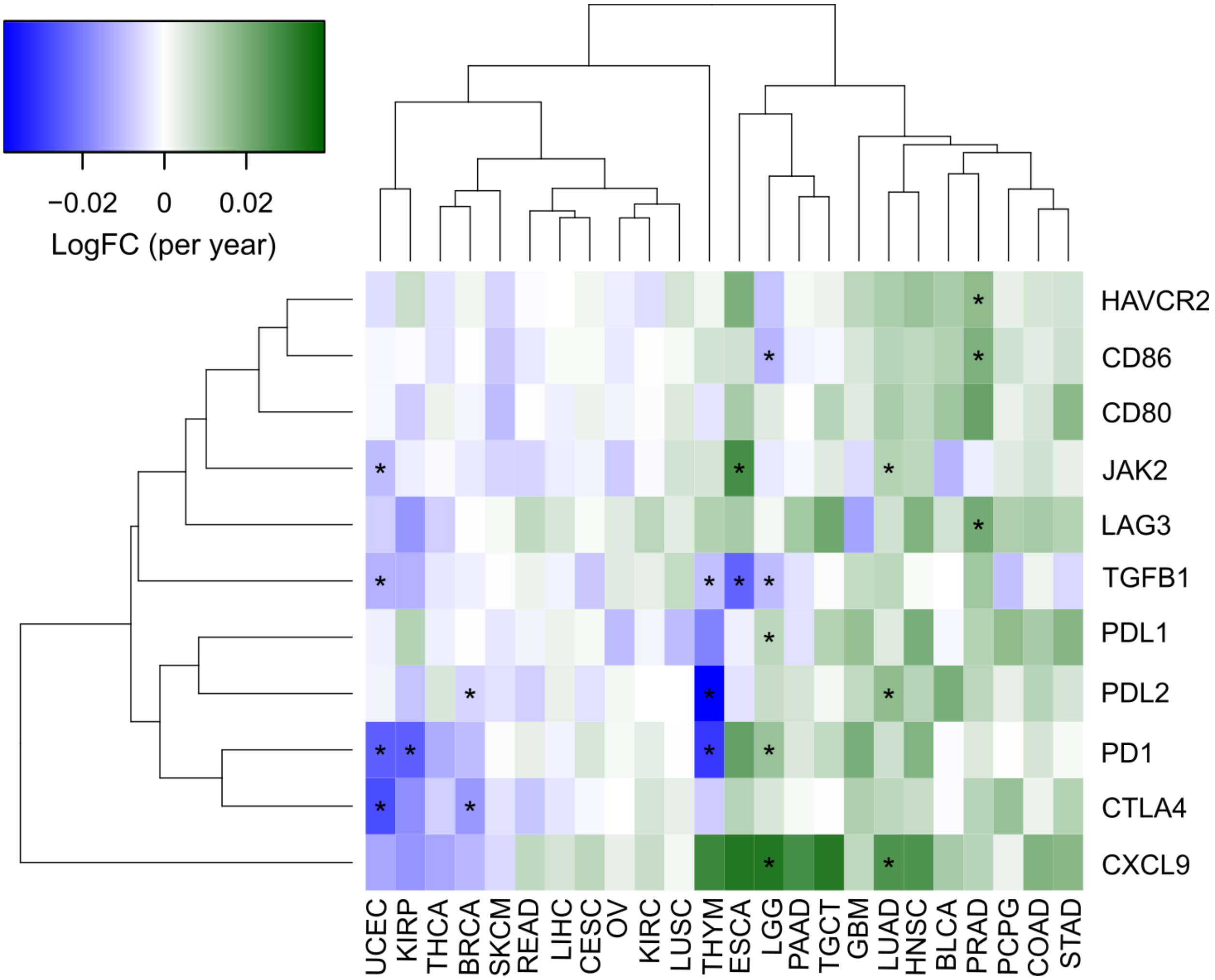

Log fold change per year of patient age from limma-voom differential expression analysis of immune checkpoint and ICB-related genes across TCGA studies. Positive log fold change indicates trends of increasing expression with increasing age. * indicates a FDR adjusted p-value less than 0.05. Relates to Figure 2.

Supplemental Figure 4 - Gene set enrichment of TGF-beta and WNT related pathways by patient age

A

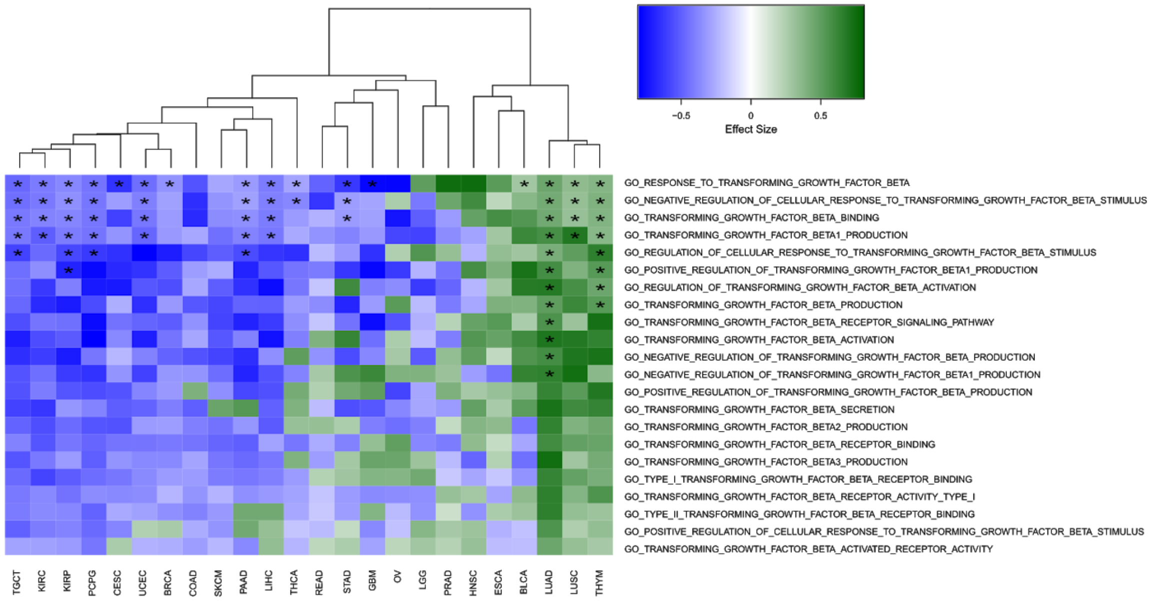

B

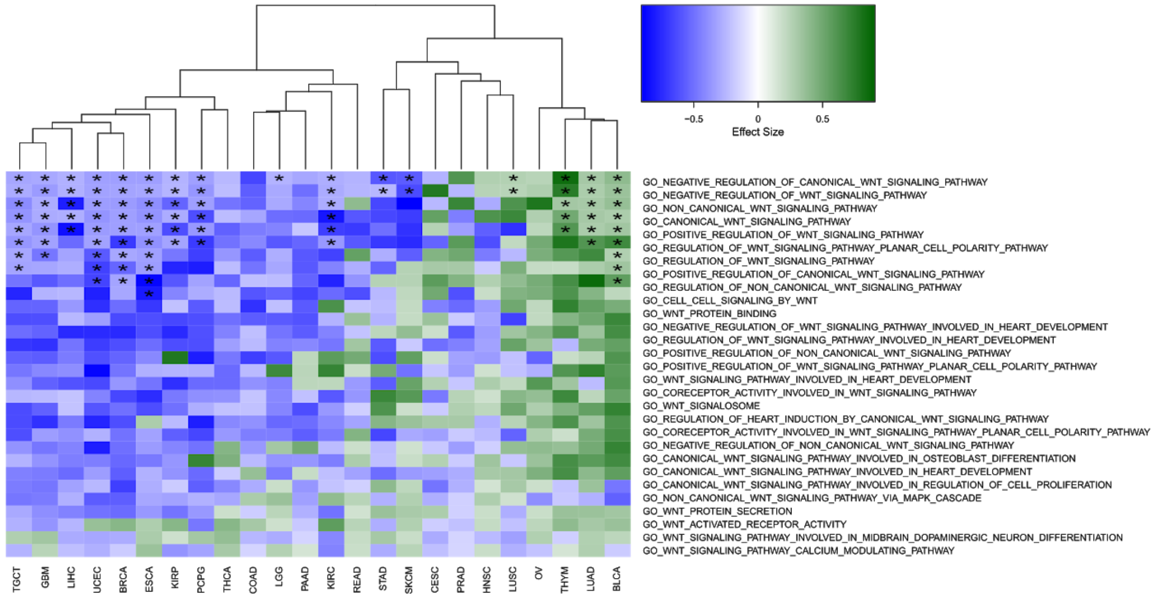

A Heatmap of estimated effect sizes from gene set enrichment of all TGFβ-related GO terms

across TCGA cancer type studies for patient age. Positive effect sizes correspond to increased enrichment with increasing age. * indicates an FDR adjusted p-value less than 0.05. **B** Heatmap of estimated effect sizes from gene set enrichment of all WNT-related GO terms across TCGA cancer type studies for patient age. Positive effect sizes correspond to increased enrichment with increasing age. * indicates an FDR adjusted p-value less than 0.05. Relates to Figure 3.

Supplemental Figure 5 - Shifts in promoter methylation of ICB genes with age in TCGA

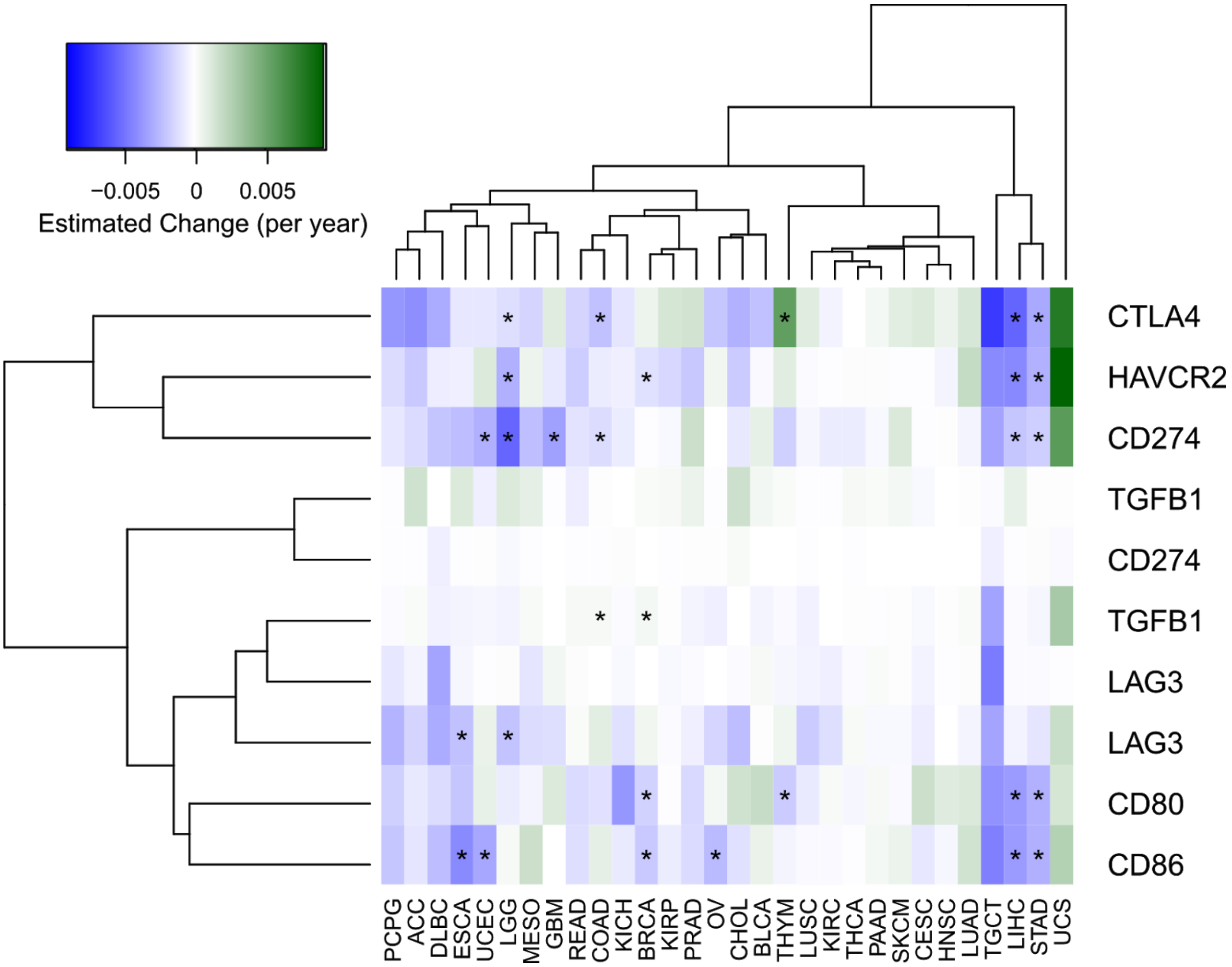

Heatmap of estimated promoter methylation change per year using a linear model with patient age across TCGA cancer type studies for ICB related genes. Positive values indicate increased methylation with increasing age. * indicates an FDR adjusted p-value less than 0.05.Relates to Table 2.

Supplemental Figure 6 - Relationship of T cell and macrophage abundance with patient age in TCGA

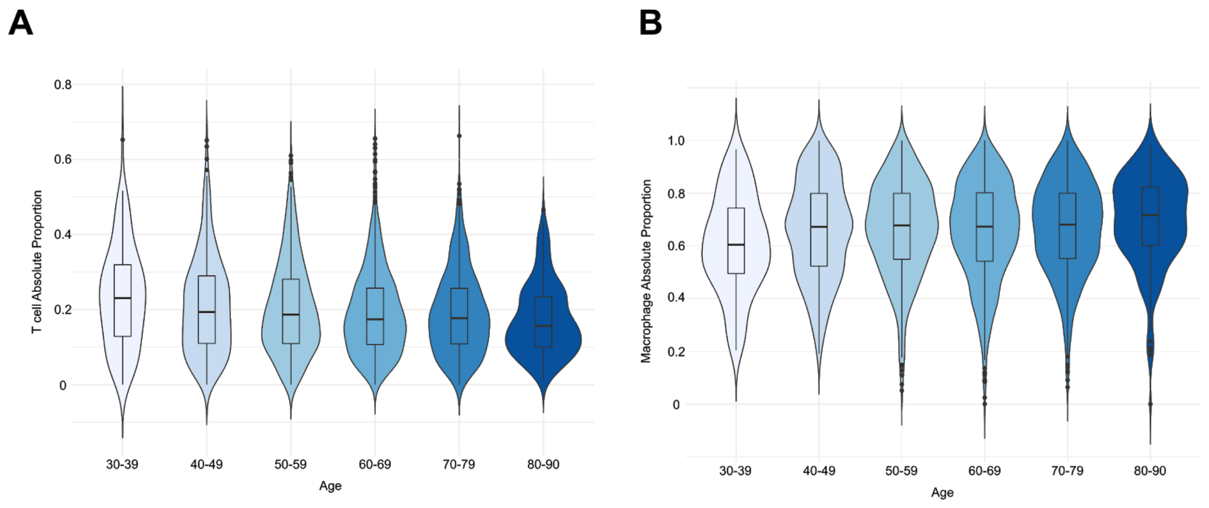

A Combined violin and boxplot of T cell absolute proportion by age-group pan-cancer in TCGA as inferred by the MIXTURE cell type deconvolution algorithm. **B** Combined violin and boxplot of macrophage absolute proportion by age-group pan-cancer in TCGA as inferred by the MIXTURE cell type deconvolution algorithm. Relates to Figure 4.

Supplemental Figure 7 -Relationship of immune cell abundance to age in GTEx

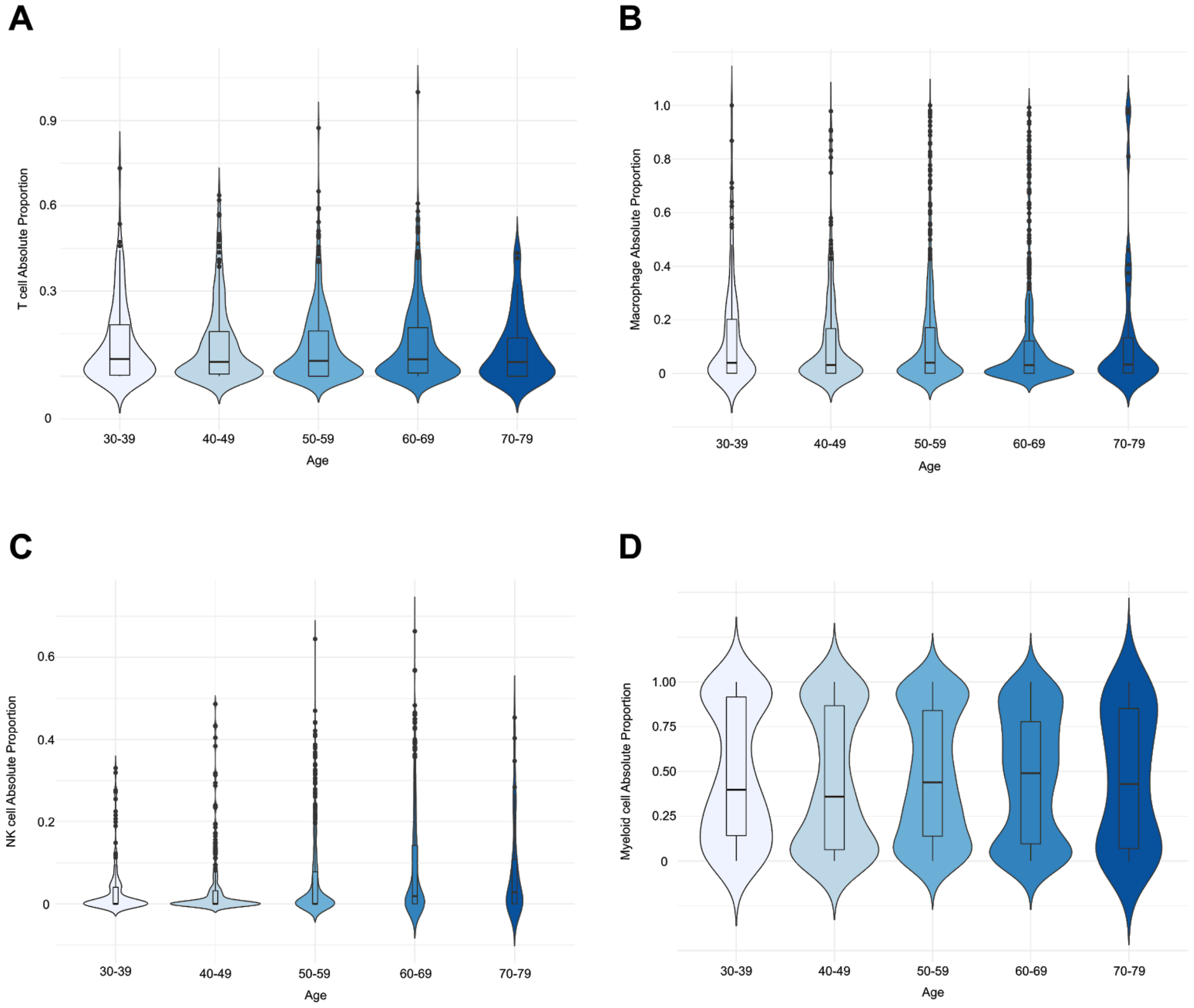

A Combined violin and boxplot of T cell absolute proportion by age-group in GTEx samples pan-tissue as inferred by the MIXTURE cell type deconvolution algorithm. **B** Combined violin and boxplot of macrophage absolute proportion by age-group in GTEx samples pan-tissue as inferred by the MIXTURE cell type deconvolution algorithm.**C** Combined violin and boxplot of NK cell absolute proportion by age-group in GTEx samples pan-tissue as inferred by the MIXTURE cell type deconvolution algorithm. **D** Combined violin and boxplot of non-macrophage myeloid cell absolute proportion by age-group in GTEx samples pan-tissue as inferred by the

MIXTURE cell type deconvolution algorithm. Relates to Figure 4.

Supplemental Figure 8 - Relationship of T cell and macrophage abundance with patient age in CLS data

A

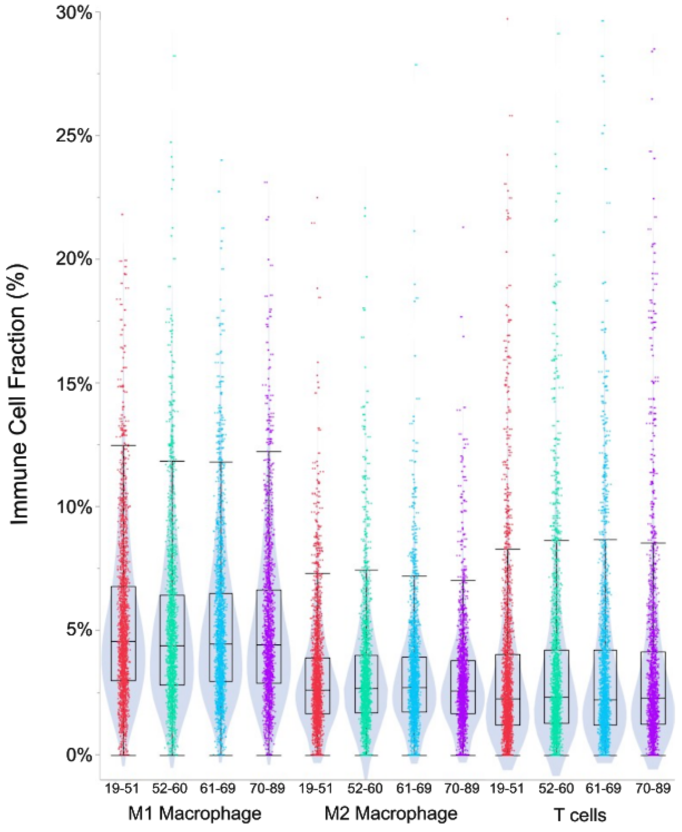

30%
25%
20%
15%
10%
5%
0%
Immune Cell Fraction (%)
19-51
52-60
61-69
70-89
19-51
52-60
61-69
70-89
19-51
52-60
61-69
70-89
M1 Macrophage
M2 Macrophage
T cells

B

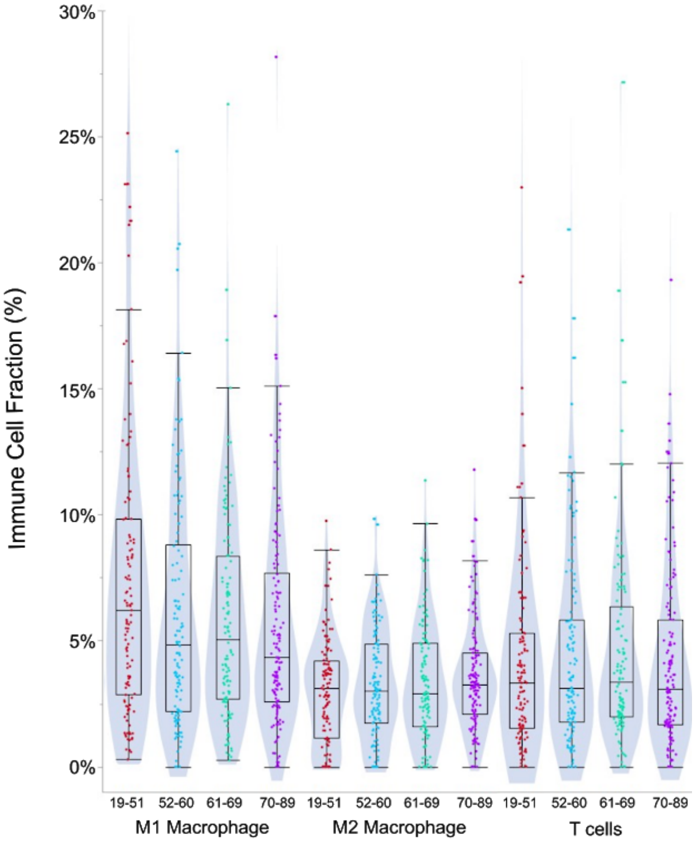

30%
25%
20%
15%
10%
5%
0%
Immune Cell Fraction (%)
19-51
52-60
61-69
70-89
19-51
52-60
61-69
70-89
19-51
52-60
61-69
70-89
M1 Macrophage
M2 Macrophage
T cells

A Combined violin and boxplots of macrophage and T cell immune cell fraction compared across age groups for 7,924 colorectal cancer patients from the CLS cohort. **B** Combined violin and boxplots of macrophage and T cell immune cell fraction compared across age groups for 517 HPV-negative head and neck cancer patients from the CLS cohort. Relates to Figure 4.

Supplemental Figure 9 - Patient age has no discernible effect on survival or progression of ICB treated RCC patients

A

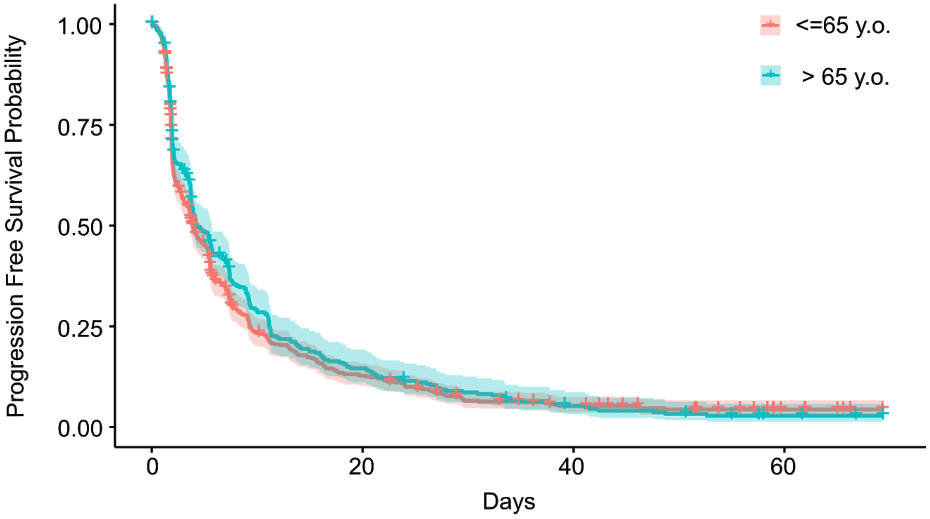

B

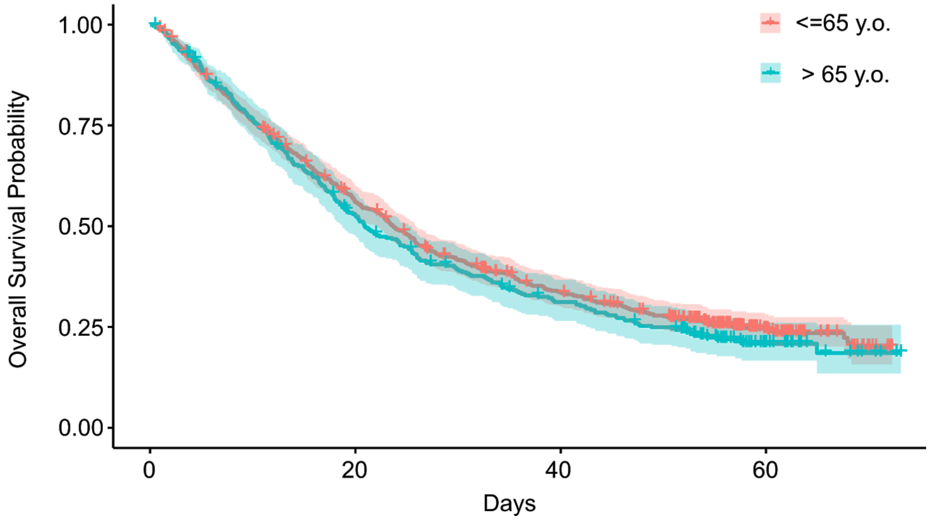

Kaplan-Meier plots of 985 renal cell carcinoma patients treated with anti-PD1/PDL1 therapies comparing patients under 65 to those 65 and older for the metrics of **A** progression free survival **B** and overall survival. No significant difference is identified between the two groups for either survival metric. Related to Figures 1-4.

Supplemental Table 1 - Differential expression of ICB genes by age in GTEx

**Gene**	**LogFC (per year)**	**t-statistic**	**p-value**	**q-value**
LAG3	0.013	14.706	<0.001	<0.001
PDL2	0.010	11.045	<0.001	<0.001
TGFB1	0.006	10.670	<0.001	<0.001
PD1	0.008	5.896	<0.001	<0.001
HAVCR2	0.005	5.526	<0.001	<0.001
PDL1	0.003	3.805	<0.001	<0.001
JAK2	-0.002	-4.740	<0.001	<0.001
CD86	0.000	0.375	0.708	0.742

Differential expression results for immune checkpoint genes and immune checkpoint related genes pan-tissue in GTEx. The results are shown for the association with age, including tissue type as a covariate. Note that LogFC is log fold change for each year of age. Relates to Figure 2.

Supplemental Table 2 - Reference of immune cell types evaluated

**Immune Cell Types**	**Immune Subtypes**
T cells	CD8 T cells, Naive CD4 T cells, Resting Memory Cd4 T cells, Activated Memory CD4 T cells, Follicular Helper T cells, Regulatory T cells, Gamma Delta T cells
Macrophages	M0 Macrophages, M1 Macrophages, M2 Macrophages
B cells	Naive B cells, Memory B cells, Plasma Cells
NK cells	Resting NK cells, Activated NK cells
Dendritic cells	Resting Dendritic cells, Activated Dendritic cells
Misc. Myeloid cells	Monocytes, Resting Mast cells, Activated Mast cells, Eosinophils, Neutrophils

Immune cell types that were evaluated in relation to age for both TCGA tumor samples and GTEx normal samples. Relates to Figure 4.

Supplemental Table 3 - Impact of immune abundance on overall patient survival

	Coefficient (per year)	Hazard Ratio	z-statistic	p-value	q-value
**T cells**	-1.25217	0.285882	-3.9169	8.97×10^{-5}	0.000538
**Macrophages**	0.694023	2.001752	3.460928	0.000538	0.001615
**B cells**	-0.52134	0.593723	-1.43318	0.151808	0.227711
**NK cells**	-1.99242	0.136365	-2.48022	0.01313	0.026261
**Dendritic cells**	0.18663	1.205181	0.368952	0.712164	0.712164
**Misc. Myeloid**	0.516746	1.676563	0.99876	0.317911	0.381493

Coefficients, Hazard ratios, p, and q-values for each immune cell term in a Cox proportional hazards model fit to predict overall patient survival, including diagnosis age, sex, cancer type, and number of years smoked as covariates. Relates to Figure 4.

Supplemental Table 4 - Immune abundance by age in GTEx

	Estimate (per year)	t-statistic	p-value	q-value
**T cells**	8.972×10^{-4}	3.497	4.831×10^{-4}	1.449×10^{-3}
**Macrophages**	4.712×10^{-5}	3.968	7.42×10^{-5}	4.45×10^{-4}
**B cells**	-6.060×10^{-4}	-1.313	0.189	0.252
**NK cells**	1.896×10^{-3}	7.838	8.554×10^{-15}	5.132×10^{-14}
**Dendritic cells**	-4.296×10^{-4}	-1.251	0.210	0.252
**Misc. Myeloid**	-1.804×10^{-3}	-2.522	0.0117	0.0235

Coefficients, statistics, p, and q-values for the diagnosis age term in the linear model fit for each immune cell type in GTEx data pan-tissues. Tissue type and sex were included as covariates for each of these models. Note that estimated coefficients are per additional year of age. Relates to Figure 4.

Supplemental Table 5 - Summary of TCGA cohort sample sizes

**TCGA Cancer Type Acronym**	**Number of Samples**	**Number of Samples After MIXTURE**
ACC	64	5
BLCA	405	122
BRCA	1056	561
CESC	270	143
CHOL	35	10
COAD	272	82
DLBC	45	0
ESCA	180	43
GBM	148	19
HNSC	507	359
KICH	61	1
KIRC	508	205
KIRP	276	69
LAML	158	0
LGG	433	9
LIHC	347	39
LUAD	479	337
LUSC	474	353
MESO	86	22

OV	292	88
PAAD	177	84
PCPG	157	0
PRAD	480	10
READ	88	23
SARC	244	125
SKCM	411	146
STAD	403	161
TGCT	74	0
THCA	433	59
THYM	116	0
UCEC	169	57
UCS	57	18
UVM	79	10

Number of RNA-seq samples from each TCGA study in the TCGA data used in this work. Relates to Figures 1-4.
